# A CD4^+^ T lymphocyte–specific TCR/GSDMD/IL-2 axis facilitates antitumor immunity

**DOI:** 10.1172/JCI191119

**Published:** 2025-08-01

**Authors:** Yihan Yao, Lingling Wang, Weiqin Jiang, Ning Wang, Mengjie Li, Wenlong Lin, Ting Zhang, Wanqiang Sheng, Xiaojian Wang

**Affiliations:** 1 Institute of Immunology and Bone Marrow Transplantation Center and; 2 Department of Colorectal Surgery, The First Affiliated Hospital, School of Medicine, Zhejiang University, Hangzhou, China.; 3 Department of Radiation Oncology, The Second Affiliated Hospital, School of Medicine, Zhejiang University, Hangzhou, China.; 4 Institute of Immunology and Department of Respiratory Disease, The First Affiliated Hospital, School of Medicine, Zhejiang University, Hangzhou, China.

**Keywords:** Immunology, Oncology, Calcium signaling, Cancer immunotherapy, T cells

## Abstract

Gasdermin (GSDM) family proteins mediate tumor pyroptosis and impact cancer progression, but other than that, their involvement in the tumor immune microenvironment remains largely unknown. Here, we show that activation of GSDMD in human tumor specimens mainly occurs in tumor-infiltrating leukocytes. Significantly, GSDMD deficiency or its inactivation in CD4^+^ T cells disabled CD8^+^ T cell–mediated antitumor immunity and caused tumor outgrowth in mice. Further study uncovered that, via inducing IL-2 production, GSDMD was required for CD4^+^ T cells to provide help to CD8^+^ T cell function. Mechanistically, GSDMD was cleaved by TCR stimulation–activated caspase-8 to form GSDMD-N pores, which enhanced Ca^2+^ influx for IL-2 induction. Moreover, GSDMD activation and function were conserved in human CD4^+^ T cells and associated with favorable prognosis and improved response to anti–PD-1 immunotherapy in colonic and pancreatic cancer. We believe this study identifies a new nonpyroptotic role of GSDMD in tumor immunity, proposing GSDMD as a potential target for cancer immunotherapy.

## Abstract 2

TCR signaling cleaved Gasdermin D to form GSDMD-N pores in the plasma membrane of CD4^+^ T cells, which triggers Ca^2+^ influx to induce the expression of IL-2 to aid CD8^+^ T-mediated tumor cell killing.

## Graphical abstract

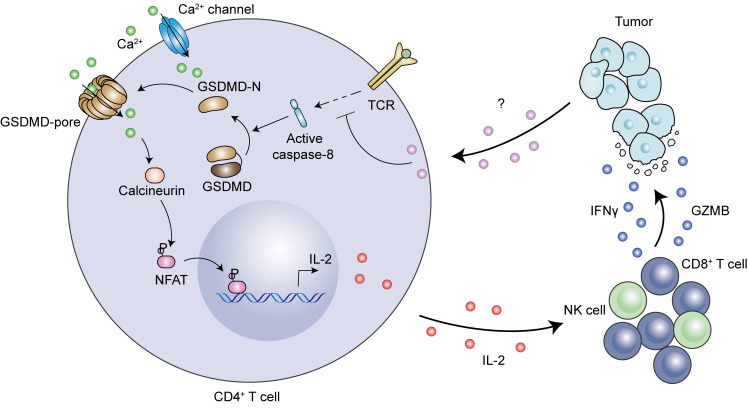

## Introduction

Immune cells are important cellular players in the tumor microenvironment (TME). Especially, T cells can exert a direct killing effect on tumor cells. The role of T cells in the TME and the underlying mechanisms have been widely reported, but how to enhance the function of cytotoxic T cells and overcome T cell exhaustion is still the focus of current investigation (1).

Although the tumor killing effect is mainly attributable to cytotoxic CD8^+^ T cells and natural killer (NK) cells, CD4^+^ T cells also play an essential role in inducing a durable antitumor immunity through multiple mechanisms (2). In addition to directly eliminating tumor cells through cytotoxicity, CD4^+^ T cells have been reported to enhance the function of other immune populations by modulating the TME. For instance, CD4^+^ T cells can produce multiple cytokines, which promote the continuous activation and survival of CD8^+^ T and NK cells as well as their differentiation into the effector phenotype (2–4). Further, CD4^+^ T cells help the maintenance of memory CD8^+^ T cells and improve the secondary response to antigen re-encounter (3). In antitumor immune response, in addition to secreting cytokines that enhance the cytotoxicity of CD8^+^ T cells, CD4^+^ T cells can also interact with other immune populations such as dendritic cells (DCs) and B cells to indirectly promote the activation and function of CD8^+^ T cells (5, 6).

IL-2, also known as the T cell growth factor, is mainly produced by activated T cells (7), although to a lesser extent by NK cells, activated DCs (8), and mast cells (9). Notably, IL-2 production by CD8^+^ T cells is far less than that by CD4^+^ T cells, and subsequently the full response of CD8^+^ cytotoxic T cells often requires help from CD4^+^ T cell–derived IL-2 (7). Particularly, in the TME, it has been reported that the source of IL-2 switches from CD8^+^ T cells to CD4^+^ T cells during tumor progression (10). IL-2 signal regulates CD8^+^ T cells at all stages of an immune response. Specifically, IL-2 promotes the proliferation and survival of naive CD8^+^ T cells upon activation, and further enhances the expression of effector molecules including IFN-γ by activated CD8^+^ T cells (11–13). In addition, IL-2 also affects the differentiation of CD8^+^ T cells into short-lived effector or long-lived memory T cells (14). These findings highlight the importance of IL-2 in antitumor T cell immunity, which is now being explored to potentiate cancer immunotherapy. Regarding IL-2 production by T cells, upon T cell receptor (TCR) activation by cognate antigens and costimulatory signals, intracellular Ca^2+^ levels are rapidly increased, leading to the activation of the calcineurin/NFAT pathway. Activated NFATs translocate into the nucleus and, together with several other transcription factors, including AP-1 and NF-κB, transcriptionally activate IL-2 expression (15). Nevertheless, additional layers of regulation of IL-2 production remain to be further explored.

The gasdermins are a family of pore-forming effector proteins that cause pyroptosis. The gasdermin (GSDM) family members comprise GSDMA, GSDMB, GSDMC, GSDMD, GSDME, and DFNB59, which display differential tissue expression. Proteolytic cleavage between N-terminal and C-terminal domains by caspases induces N-terminal domain oligomerization, which forms pores in the cell membrane and subsequently induces cell death (16). Increasing evidence shows that gasdermin family proteins are expressed in tumor cells, and their activation leads to cell pyroptosis and inhibits tumor growth (17–20). Gasdermins, especially GSDMD and GSDMB, are also expressed in immune cells (16), but their functions have just begun to be explored. A recent study reported that GSDMD in myeloid cells inhibited cGAS-dependent antitumor immunity in response to anti–PD-L1 treatment (21). Other than that, the role of gasdermins in a variety of immune cell populations pertaining to tumor immunity remains elusive.

In this study, we identified an indispensable role of GSDMD in CD4^+^ T cells for antitumor immunity. Upon TCR activation, caspase-8 in CD4^+^ T cells cleaves GSDMD and triggers the formation of the GSDMD-N pores, which allows extracellular Ca^2+^ influx, an essential event for IL-2 expression. GSDMD-mediated IL-2 production by CD4^+^ T cells is required for the activation and effector function of both CD8^+^ T and NK cells. Importantly, GSDMD activation in intratumoral CD4^+^ T lymphocytes correlates with survival benefit and immunotherapy efficacy in colorectal cancer and pancreatic adenocarcinoma. Our study demonstrates a CD4^+^ T cell–specific role of GSDMD in antitumor immunity, independent of its canonical function in pyroptosis, which may raise a new perspective for cancer immunotherapy.

## Results

### GSDMD is required for immune cells to exert an antitumor effect.

**Figure 1:**
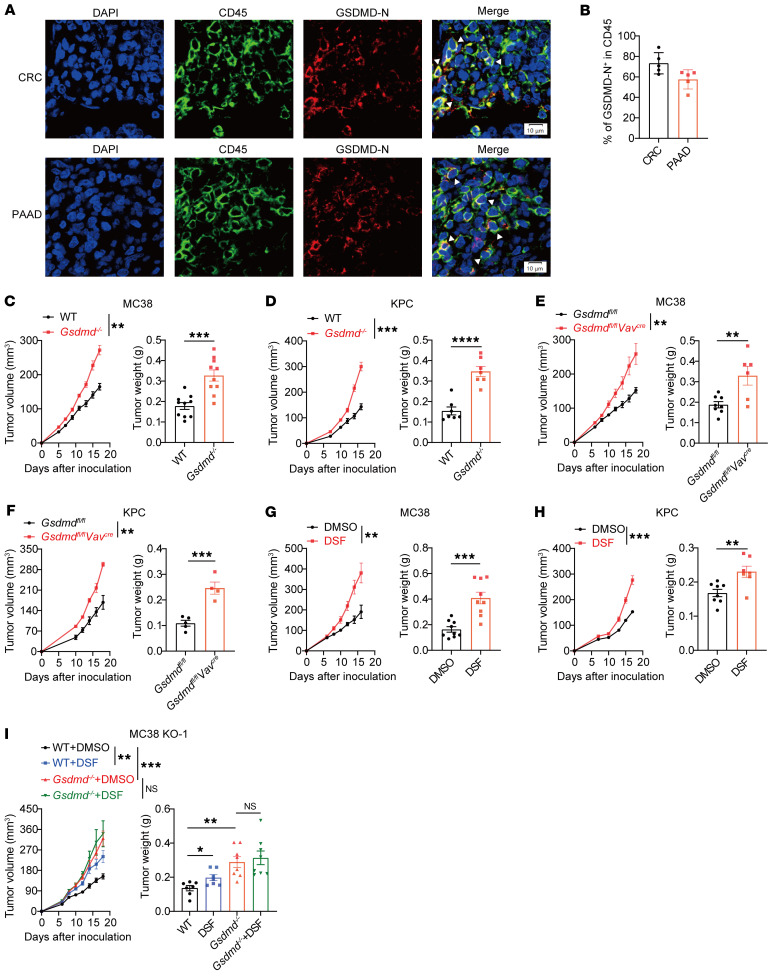
GSDMD deficiency in immune cells promotes tumor growth. (**A** and **B**) Immunofluorescence staining of GSDMD-N (red) and CD45 (green) in tumor tissues from colorectal or pancreatic cancer patients (**A**). The percentages of GSDMD-N^+^ cells among CD45^+^ cells were quantified from 5 independent fields of view within CRC and PAAD tumor tissues (**B**). Scale bars: 10 μm. The white arrowheads indicate GSDMD-N– and CD45-coexpressing cells. CRC, colorectal cancer; PAAD, pancreatic adenocarcinoma. (**C** and **D**) Tumor growth curves (left) and tumor weight (right) of WT and *Gsdmd^–/–^* mice subcutaneously inoculated with MC38 (**C**, *n* = 10 per group) or KPC tumor cells (**D**, *n* = 7 per group). (**E** and **F**) Tumor growth curves (left) and tumor weight (right) of *Gsdmd^fl/fl^* and *Gsdmd^fl/fl^ Vav^cre^* mice subcutaneously inoculated with MC38 (**E**, *n* = 6–8 per group) or KPC tumor cells (**F**, *n* = 4–5 per group). (**G** and **H**) Tumor growth curves (left) and tumor weight (right) of WT mice subcutaneously inoculated with MC38 (**G**, *n* = 9 per group) or KPC tumor cells (**H**, *n* = 7–8 per group) and treated with DMSO or disulfiram (DSF). (**I**) Tumor growth curves (left) and tumor weight (right) of WT and *Gsdmd^–/–^* mice inoculated with *Gsdmd^–/–^* MC38 tumor cells and treated with DMSO or DSF (*n* = 7–8 per group). Data are presented as mean ± SEM (**B**–**I**) and are representative of at least 2 independent experiments. *P < 0.05; **P < 0.01; ***P < 0.001; ****P < 0.0001; NS, not significant; as determined by unpaired 2-tailed Student’s *t* tests for tumor weights in **C**–**H** and 1-way ANOVA for tumor weights in **I** or 2-way ANOVA for tumor growth curves.

To examine the expression pattern of GSDMD in the TME, we performed immunofluorescence with tumor specimens from colorectal cancer and pancreatic adenocarcinoma patients. We observed the expression of GSDMD in both tumor cells and immune cells (Supplemental Figure 1A; supplemental material available online with this article; https://doi.org/10.1172/JCI191119DS1), with the majority of tumor-infiltrating immune cells expressing GSDMD (Supplemental Figure 1, B and C). Importantly, GSDMD was localized on the cell membrane of immune cells, indicating an active form of GSDMD (Supplemental Figure 1B). Consistently, we found an accumulation of cleaved GSDMD-N on immune cell membrane using cleaved GSDMD-N–specific antibodies that did not recognize full-length GSDMD or cleaved GSDMD-C (Figure 1, A and B). In contrast, GSDMD activation was infrequently observed in tumor cells (Supplemental Figure 1D). These results highlight the frequent activation of GSDMD in tumor-infiltrating immune cells, as opposed to tumor parenchymal cells.

To investigate the impact of GSDMD activation in immune cells on tumor immunity, we used *Gsdmd*-conventional-knockout (*Gsdmd^–/–^*) mice in syngeneic tumor models. In line with a previous study (22), flow cytometry analysis showed that GSDMD was commonly expressed in lymphocytes and myeloid cells (Supplemental Figure 1E). However, GSDMD knockout had no overt effect on the cellular compositions in the spleen, lymph nodes, and peripheral blood (Supplemental Figure 1, F–H). We found that subcutaneously inoculated colonic MC38 tumors grew faster in the *Gsdmd^–/–^* mice than in wild-type (WT) mice (Figure 1C). Similar results were observed in a pancreatic KPC tumor model (Figure 1D) and a melanoma B16-OVA tumor model (Supplemental Figure 1I). Consistently, *Gsdmd^fl/fl^ Vav^cre^* mice, with specific depletion of GSDMD in hematopoietic cells, also displayed accelerated tumor growth when inoculated with MC38 or KPC tumors (Figure 1, E and F). These results suggest that GSDMD expression in immune cells exerts a tumor growth–inhibitory effect.

Disulfiram (DSF), an FDA-approved medicine for the treatment of alcohol dependence (23), has recently been identified as an inhibitor of GSDMD, acting via inhibition of GSDMD-N oligomerization (24). Treating WT mice with DSF significantly promoted tumor growth in both MC38 and KPC models (Figure 1, G and H), whereas this tumor-promoting effect was minimized in *Gsdmd^–/–^* mice (Supplemental Figure 1J). To further characterize the cellular target of in vivo DSF treatment, we generated GSDMD-knockout MC38 cells (Supplemental Figure 1K) and showed that, in contrast to the diminished tumor-promoting effect of DSF treatment in the *Gsdmd^–/–^* mice, DSF treatment retained the ability to promote the growth of GSDMD-deficient MC38 tumors in WT mice (Figure 1I). Thus, DSF treatment targets GSDMD activation particularly in immune cells, leading to tumor progression. In addition, another GSDMD inhibitor, dimethyl fumarate (DMF) (25), also promoted MC38 tumor growth in WT mice (Supplemental Figure 1L). Collectively, our data suggest that GSDMD activation is crucial for immune cells to restrain tumor growth.

### GSDMD inactivation compromises T cell–mediated antitumor immunity.

**Figure 2:**
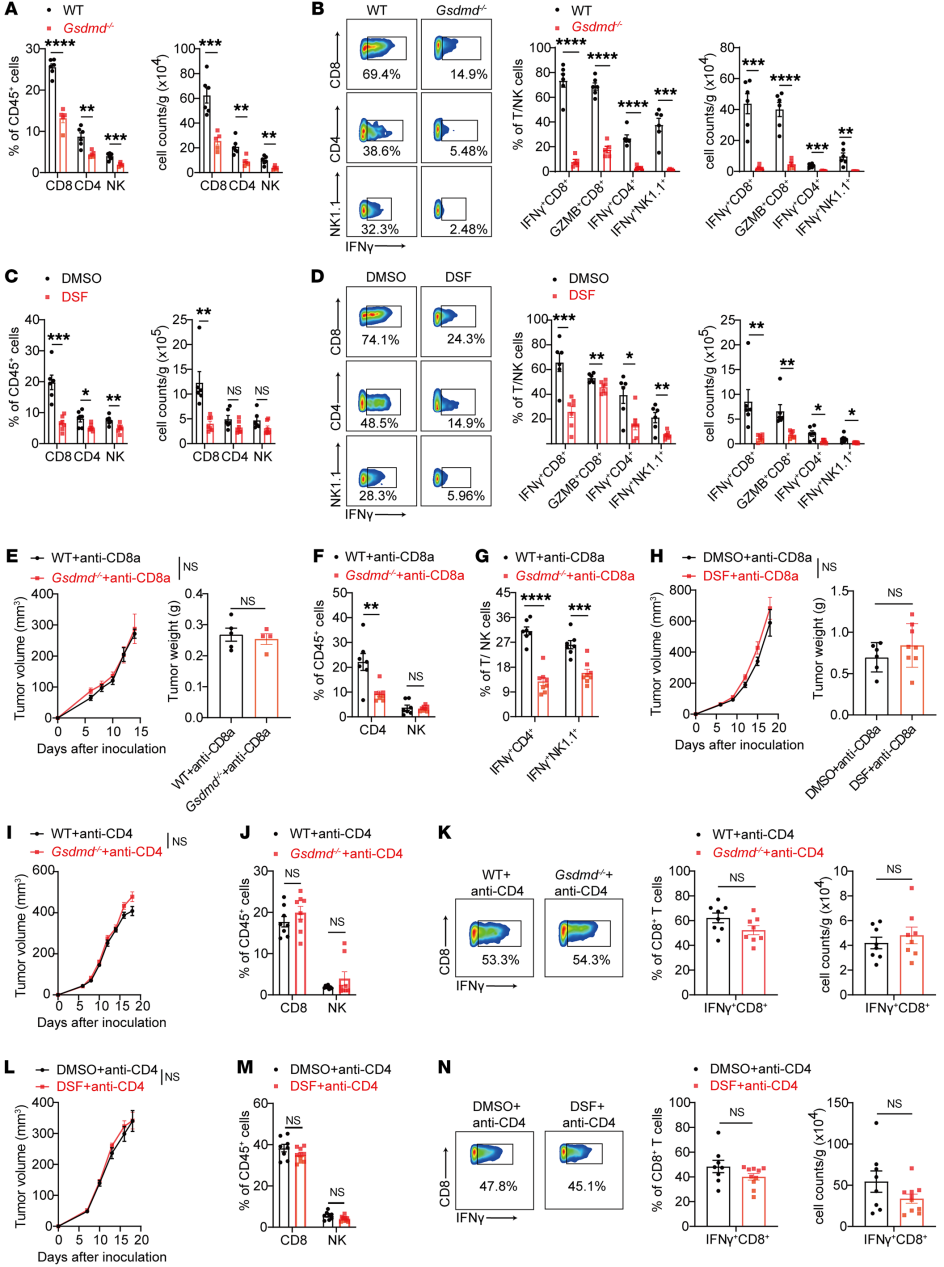
GSDMD inactivation impairs antitumor immunity in a T cell–dependent manner. (**A** and **B**) Flow cytometry analysis of percentages (left) and cell numbers (right) of tumor-infiltrating lymphocytes (TILs) (**A**) and expression of IFN-γ and granzyme B by TILs (**B**) isolated from MC38 tumor–bearing WT (*n* = 6) and *Gsdmd^–/–^* (*n* = 5) mice on day 18 after tumor inoculation. (**C** and **D**) Flow cytometry analysis of lymphocyte infiltration (**C**) and effector molecule expression (**D**) in MC38 tumors implanted in WT mice and treated with DMSO (*n* = 6) or DSF (*n* = 7). (**E**) Tumor growth curves (left) and tumor weight (right) of MC38 tumors in WT (*n* = 5) and *Gsdmd^–/–^* (*n* = 4) mice treated with CD8α-depleting antibodies. (**F** and **G**) Flow cytometry analysis of percentages of CD4^+^ and NK TILs (**F**) and IFN-γ expression by TILs (**G**) in MC38 tumors isolated from WT (*n* = 7) and *Gsdmd^–/–^* (*n* = 8) mice treated with CD8α-depleting antibodies. (**H**) Tumor growth curves (left) and tumor weights (right) of MC38 tumors in WT mice injected with CD8α-depleting antibodies and treated with DMSO (*n* = 6) or DSF (*n* = 8). (**I**–**K**) Tumor growth curves of MC38 tumors in WT and *Gsdmd^–/–^* mice injected with CD4-depleting antibodies (**I**, *n* = 8 per group), and flow cytometry analysis of percentages of CD8^+^ and NK TILs (**J**) and IFN-γ expression by CD8^+^ TILs (**K**). (**L**–**N**) Tumor growth curves (**L**) of MC38 tumors in WT mice injected with CD4-depleting antibodies and treated with DMSO (*n* = 8) or DSF (*n* = 10), and flow cytometry analysis of percentages of CD8^+^ and NK TILs (**M**) and IFN-γ expression by CD8^+^ TILs (**N**). Data are presented as mean ± SEM and are representative of at least 2 independent experiments (**A**–**N**). *P < 0.05; **P < 0.01; ***P < 0.001; ****P < 0.0001; NS, not significant; as determined by 2-way ANOVA for tumor growth curves or unpaired 2-tailed Student’s *t* tests for TILs.

To explore how GSDMD inactivation affected antitumor immunity, we performed flow cytometry analyses of tumor-infiltrating leukocytes (TILs) on day 18 after MC38 implantation (Supplemental Figure 2A). Tumors from *Gsdmd^–/–^* mice exhibited much less infiltration of antitumor lymphocytes, including CD8^+^ and CD4^+^ T cells as well as NK cells (Figure 2A), while other immune populations were largely unaffected by GSDMD depletion (Supplemental Figure 2B). This reduction in lymphocyte infiltration was associated with decreased proliferation, as indicated by Ki67 staining, whereas cell survival and the CXCL9/10-CXCR3 axis, critical for lymphocyte recruitment to tumors (26, 27), remained unchanged (Supplemental Figure 2, C–H). Further analysis revealed that infiltrating lymphocytes in *Gsdmd^–/–^* mice exhibited severe functional defects, evidenced by the diminished expression of IFN-γ and granzyme B (Figure 2B). Notably, this dysfunction was not due to exhaustion, as CD8^+^ TILs showed no significant differences in the expression of PD-1, CD39, and TIM3, nor in the frequency of terminally exhausted (PD-1^+^CD39^+^TIM3^+^) CD8^+^ T cells between 2 groups (Supplemental Figure 2, I and J). Consistent with genetic data, GSDMD inhibition by DSF treatment in MC38 tumor–bearing mice also reduced the infiltration and function of T cells and NK cells (Figure 2, C and D), while changes in myeloid cells were minimal (Supplemental Figure 2K). The suppressed effector function of tumor-infiltrating lymphocytes was also observed with DMF treatment (Supplemental Figure 2L). The phenotypes of tumor growth advantage and lymphocyte function impairment in GSDMD-deficient mice were recapitulated in the KPC and B16-OVA tumor models (Supplemental Figure 2, M–P). Moreover, specific depletion of GSDMD in hematopoietic cells also suppressed the dysfunction of T cells and NK cells in the MC38 tumor model (Supplemental Figure 2Q).

To determine the effector cells, whose functional impairment by GSDMD inactivation was responsible for the observed tumor growth acceleration, we used depleting antibodies to target specific immune populations. Depletion of CD8^+^ T cells abrogated the tumor growth difference between WT and *Gsdmd^–/–^* mice (Figure 2E and Supplemental Figure 2R), despite the functional impairment in GSDMD-deficient CD4^+^ T cells and NK cells (Figure 2, F and G, and Supplemental Figure 2S). Comparable tumor growth was also observed between vehicle- and DSF-treated mice after CD8^+^ T cell depletion (Figure 2H). Further, CD4^+^ T cell depletion by specific antibodies also abrogated the tumor growth difference between WT and *Gsdmd^–/–^* mice (Figure 2I and Supplemental Figure 2R), and, notably, comparable tumor infiltration and IFN-γ production by CD8^+^ T cells were detected (Figure 2, J and K). Consistent results were observed in the DSF treatment experiment (Figure 2, L–N). In contrast to the T cell depletion, depletion of NK cells by specific antibodies affected neither the difference in tumor growth nor the T cell phenotypes (Supplemental Figure 2, T–V). To evaluate the involvement of macrophages, we injected clodronate liposomes intratumorally to deplete tumor-associated macrophages (TAMs) (Supplemental Figure 2W). Flow cytometry analyses showed that IFN-γ production by intratumoral T cells and NK cells was still lower in the *Gsdmd^–/–^* mice compared with WT mice in the absence of TAMs (Supplemental Figure 2X). As a crucial executor of pyroptosis and a downstream target of inflammasomes, GSDMD is essential for the secretion of IL-18 and IL-1β (16), both of which have been reported to promote antitumor immunity by facilitating the maintenance of effector CD8^+^ T cells (28). Although the levels of IL-18 and IL-1β were expectedly reduced in the TME by *Gsdmd* knockout or DSF treatment (Supplemental Figure 2Y), neutralizing IL-18 and IL-1β did not change the difference in tumor growth between *Gsdmd^–/–^* or DSF-treated mice and WT untreated mice (Supplemental Figure 2Z), nor the difference in lymphocyte infiltration and function (Supplemental Figure 2, AA and AB). Together, the above results suggest that GSDMD inactivation compromises the antitumor immunity mediated by T cells, in a manner independent of NK cells and macrophages as well as IL-18 and IL-1β.

### GSDMD deficiency in CD4^+^ T cells blunts CD8^+^ T cell function.

**Figure 3:**
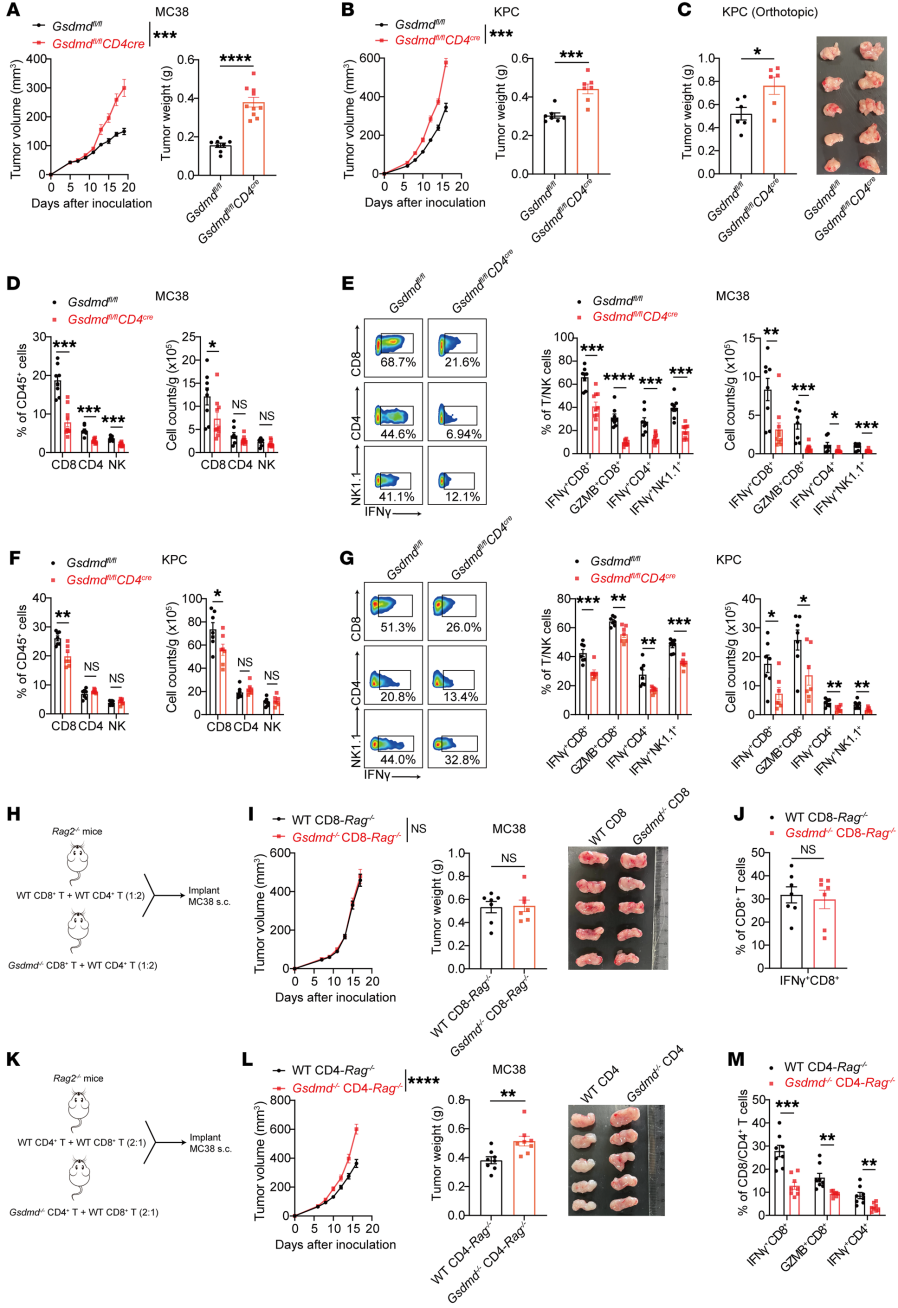
Deletion of GSDMD in CD4^+^ T cells leads to impaired CD8^+^ T cell function. (**A** and **B**) Tumor growth curves (left) and tumor weights (right) of *Gsdmd^fl/fl^* and *Gsdmd^fl/fl^ CD4^cre^* mice 18 days after subcutaneous inoculation with MC38 (**A**, *n* = 8–10 per group) or KPC tumor cells (**B**, *n* = 7 per group). (**C**) Tumor weights (left) and representative tumor images (right) of *Gsdmd^fl/fl^* and *Gsdmd^fl/fl^ CD4^cre^* mice 18 days after orthotopic injection with KPC cells into the pancreas (*n* = 6 per group). (**D**–**G**) Percentages (left) and cell numbers (right) of CD8^+^, CD4^+^, and NK TILs (**D** and **F**) and expression of IFN-γ and granzyme B by TILs (**E** and **G**) in MC38 (**D** and **E**) and KPC tumors (**F** and **G**) harvested from mice in **A** and **B**. (**H**–**J**) Experimental design (**H**) and tumor growth curves (**I**, left), weights (**I**, middle), and representative images (**I**, right) of MC38 tumors implanted in *Rag2^–/–^* mice reconstituted with WT or *Gsdmd^–/–^* CD8^+^ T cells plus WT CD4^+^ T cells (*n* = 7 per group). Percentages of IFN-γ–expressing CD8^+^ TILs were analyzed by flow cytometry (**J**). (**K**–**M**) Experimental design (**K**) and tumor growth curves (**L**, left), weights (**L**, middle), and representative images (**L**, right) of MC38 tumors implanted in *Rag2^–/–^* mice reconstituted with WT or *Gsdmd^–/–^* CD4^+^ T cells plus WT CD8^+^ T cells (*n* = 8 per group). The expression of IFN-γ and granzyme B (GZMB) by CD8^+^ and CD4^+^ TILs was analyzed (**M**). Data are presented as mean ± SEM (**A**–**G**, **I**, **J**, **L**, and **M**) and are representative of at least 2 independent experiments (**A**–**G**). *P < 0.05; **P < 0.01; ***P < 0.001; ****P < 0.0001; NS, not significant; as determined by 2-way ANOVA for tumor growth curves or unpaired 2-tailed Student’s *t* tests calculated for others.

To confirm the importance of T cell–intrinsic GSDMD for antitumor T cell immunity, we used *Gsdmd*-conditional-knockout (*Gsdmd^fl/fl^ CD4^cre^*) mice to ablate GSDMD in T cells, including CD4^+^ and CD8^+^ T cells (Supplemental Figure 3A). T cell–specific depletion of GSDMD resulted in faster tumor growth in both MC38 and KPC subcutaneous tumor models (Figure 3, A and B) and the KPC orthotopic tumor model (Figure 3C), consistent with the earlier observations in conventional-knockout mice (Figure 1, C and D). As expected, CD8^+^ T cells exhibited decreased intratumoral infiltration in the *Gsdmd^fl/fl^ CD4^cre^* mice, and both T cells and NK cells were functionally impaired in terms of IFN-γ and granzyme B expression (Figure 3, D–G, and Supplemental Figure 3, B and C), whereas changes in intratumoral myeloid cells were marginal (Supplemental Figure 3, B, D, and E). Notably, T cell development and maturation was not affected by the loss of GSDMD (Supplemental Figure 3, F–H), consistent with a previous report (22). In line with the myeloid depletion results (Supplemental Figure 2X) and a recent report (21), specific deletion of GSDMD in myeloid cells had no impact on tumor growth (Supplemental Figure 3, I and J). Our data thus demonstrate the necessity of GSDMD in T cells for their antitumor immunity.

To further distinguish the role of GSDMD in CD4^+^ versus CD8^+^ T cells, we conducted adoptive transfer experiments with *Rag2^–/–^* mice. First, we cotransferred WT CD45.1 CD8^+^ T cells and *Gsdmd^–/–^* CD45.2 CD8^+^ T cells into *Rag2^–/–^* recipient mice followed by MC38 implantation (Supplemental Figure 3K). Surprisingly, the effector function of *Gsdmd^–/–^* CD8^+^ TILs was observed to be similar to that of the WT counterparts (Supplemental Figure 3L). Second, we cotransferred WT or *Gsdmd^–/–^* CD8^+^ T cells, respectively, with WT CD4^+^ T cells into 2 groups of *Rag2^–/–^* mice (Figure 3H). These two groups showed comparable tumor volumes and CD8^+^ T cell function (Figure 3, I and J). Third, WT or *Gsdmd^–/–^* CD4^+^ T cells, respectively, with WT CD8^+^ T cells were cotransferred into *Rag2^–/–^* mice (Figure 3K). In this experiment, mice with cotransferred *Gsdmd^–/–^* CD4^+^ T cells and WT CD8^+^ T cells had an increased tumor burden (Figure 3L) and reduced T cell function compared with mice receiving both WT T cells (Figure 3M). These data, together with the earlier demonstration that CD4^+^ T cell depletion normalized the functional defects in *Gsdmd^–/–^* CD8^+^ T cells (Figure 2K), strongly suggest that loss of GSDMD in CD4^+^ T cells dampens the antitumor effect of CD8^+^ T cells, whereas GSDMD expression in CD8^+^ T cells seems dispensable for their effector function.

### GSDMD potentiates CD4^+^ T cell help to CD8^+^ T cell immunity via induction of IL-2 production.

**Figure 4:**
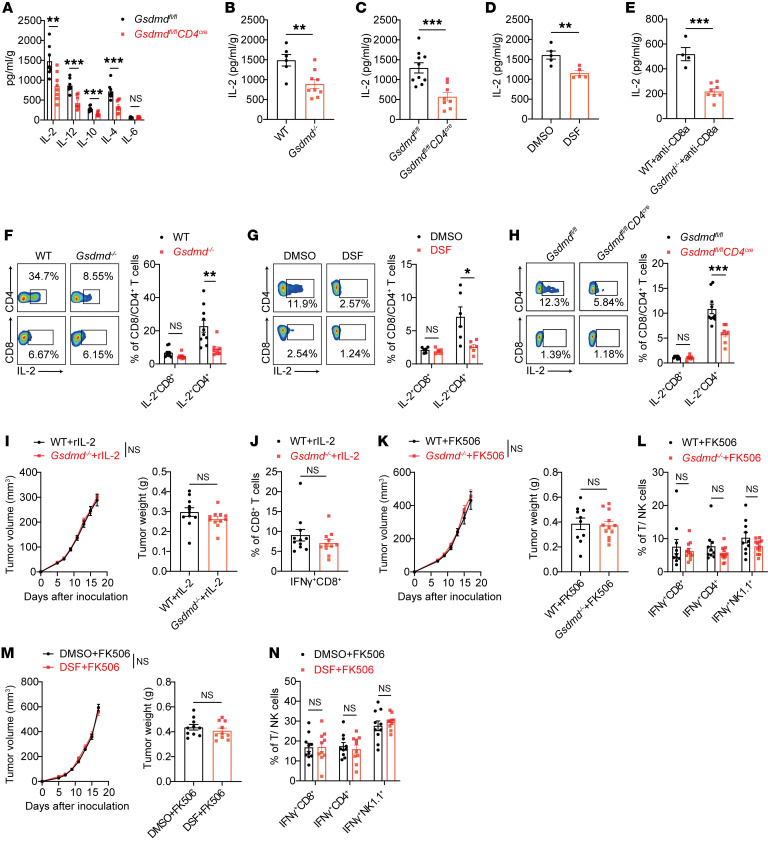
GSDMD in CD4^+^ T cells promotes antitumor immunity by increasing IL-2 production. (**A**) Levels of the indicated cytokines in supernatant of MC38 tumors isolated from *Gsdmd^fl/fl^* and *Gsdmd^fl/fl^ CD4^cre^* mice, determined by LEGENDplex (BioLegend) (*n* = 8 per group). (**B**–**E**) Quantifications of IL-2 by ELISA in supernatant of MC38 tumors isolated from WT (*n* = 6) and *Gsdmd^–/–^* (*n* = 9) mice (**B**), *Gsdmd^fl/fl^* (*n* = 10) and *Gsdmd^fl/fl^ CD4^cre^* (*n* = 8) mice (**C**), WT mice treated with DMSO or DSF (**D**, *n* = 5 per group), or WT (*n* = 4) and *Gsdmd^–/–^* (*n* = 8) mice treated with CD8α-depleting antibodies (**E**). (**F**–**H**) Percentages of IL-2–expressing CD8^+^ and CD4^+^ TILs in MC38 tumors implanted in WT (*n* = 10) and *Gsdmd^–/–^* (*n* = 9) mice (**F**), WT mice treated with DMSO or DSF (*n* = 6 per group) (**G**), or *Gsdmd^fl/fl^* (*n* = 10) and *Gsdmd^fl/fl^ CD4^cre^* (*n* = 8) mice (**H**), analyzed by flow cytometry. (**I** and **J**) Tumor growth curves (left) and tumor weights (right) of WT and *Gsdmd^–/–^* mice inoculated with MC38 tumor cells and treated with recombinant IL-2 (**I**, *n* = 11 per group). Percentages of IFN-γ–expressing CD8^+^ TILs were assessed (**J**). (**K** and **L**) Tumor growth curves (left) and tumor weights (right) of MC38 tumors in WT (*n* = 10) and *Gsdmd^–/–^* (*n* = 12) mice treated with FK506 (**K**). Percentages of IFN-γ–expressing TILs were assessed (**L**). (**M** and **N**) Tumor growth curves (left) and tumor weights (right) of MC38 tumors in WT mice cotreated with DMSO or DSF and FK506 (**M**, *n* = 10 per group). Percentages of IFN-γ–expressing TILs were assessed (**N**). Data are presented as mean ± SEM and are representative of at least 2 independent experiments (**A**–**N**). *P < 0.05; **P < 0.01; ***P < 0.001; NS, not significant; as determined by 2-way ANOVA for tumor growth curves or unpaired 2-tailed Student’s *t* tests for others.

We next sought to investigate how GSDMD regulated CD4^+^ T cells, which provide critical help to CD8^+^ T cell immunity. Considering that the effector function of CD4^+^ T cells largely relies on cytokine production, we detected the cytokine milieus in the TME. Multiple cytokines, including IL-2, IL-12, IL-10, and IL-4, showed significant reduction in tumor tissues derived from *Gsdmd^–/–^* mice compared with WT mice (Figure 4A). As IL-12, IL-10, and IL-4 are closely related to CD4^+^ T cell subset differentiation, this prompted us to test whether GSDMD affected the differentiation of CD4^+^ T cells. Nevertheless, in vitro differentiation of naive CD4^+^ T cells into Th1, Th2, or Treg subpopulations was not affected by the loss of GSDMD (Supplemental Figure 4A). In addition, TCR stimulation induced comparable activation and proliferation of CD4^+^ T cells from WT and *Gsdmd*^–*/*–^ mice, as evidenced by similar upregulation of CD69, CD25, and CD44, and equivalent CFSE dilution (Supplemental Figure 4, B and C).

IL-2 is known as a T cell growth factor and also can induce IFN-γ expression in T cells and NK cells (11, 12). We then questioned whether GSDMD deficiency in CD4^+^ T cells primarily suppressed IL-2 production, which subsequently led to the reduced intratumoral infiltration and IFN-γ production by both T and NK cells in the earlier results. To test this, we first confirmed that GSDMD deficiency in T cells, and its inactivation by DSF treatment, both decreased the IL-2 levels in the TME of multiple tumor models, regardless of the presence or absence of CD8^+^ T cells (Figure 4, B–E, and Supplemental Figure 4, D–G). Consistently, flow cytometry results demonstrated that CD4^+^ T cells were the main producers of IL-2 in the TME, whereas CD8^+^ T cells showed limited IL-2 expression (Figure 4, F–H, and Supplemental Figure 4, H–J), in line with the previous report (10). Upon the loss or inactivation of GSDMD, the frequency of IL-2–producing CD4^+^ T cells was significantly reduced, in stark contrast to the unchanged IL-2 expression by CD8^+^ T cells (Figure 4, F–H, and Supplemental Figure 4, H–J). Importantly, when the reduced IL-2 levels were overridden by supplementation with murine recombinant IL-2, the difference in IFN production by WT and *Gsdmd^–/–^* CD8^+^ TILs was diminished, which was associated with the comparable tumor growth between the two groups (Figure 4, I and J). Conversely, eliminating IL-2 expression using tacrolimus (FK506), an inhibitor of calcineurin (29), also diminished the difference in tumor growth (Figure 4K and Supplemental Figure 4K), as well as the IFN production by WT and *Gsdmd^–/–^* lymphocytes, including CD4^+^ T, CD8^+^ T, and NK cells (Figure 4L and Supplemental Figure 4L). These observations were recapitulated when DSF treatment was substituted for GSDMD depletion (Figure 4, M and N, and Supplemental Figure 4, M and N). Collectively, these results demonstrate that inactivation of GSDMD in CD4^+^ T cells dampens IL-2 production and subsequently disables CD4^+^ T cell help to CD8^+^ T cell immunity.

### GSDMD-N pores are critical for IL-2 induction by mediating Ca^2+^ influx in CD4^+^ T cells.

**Figure 5:**
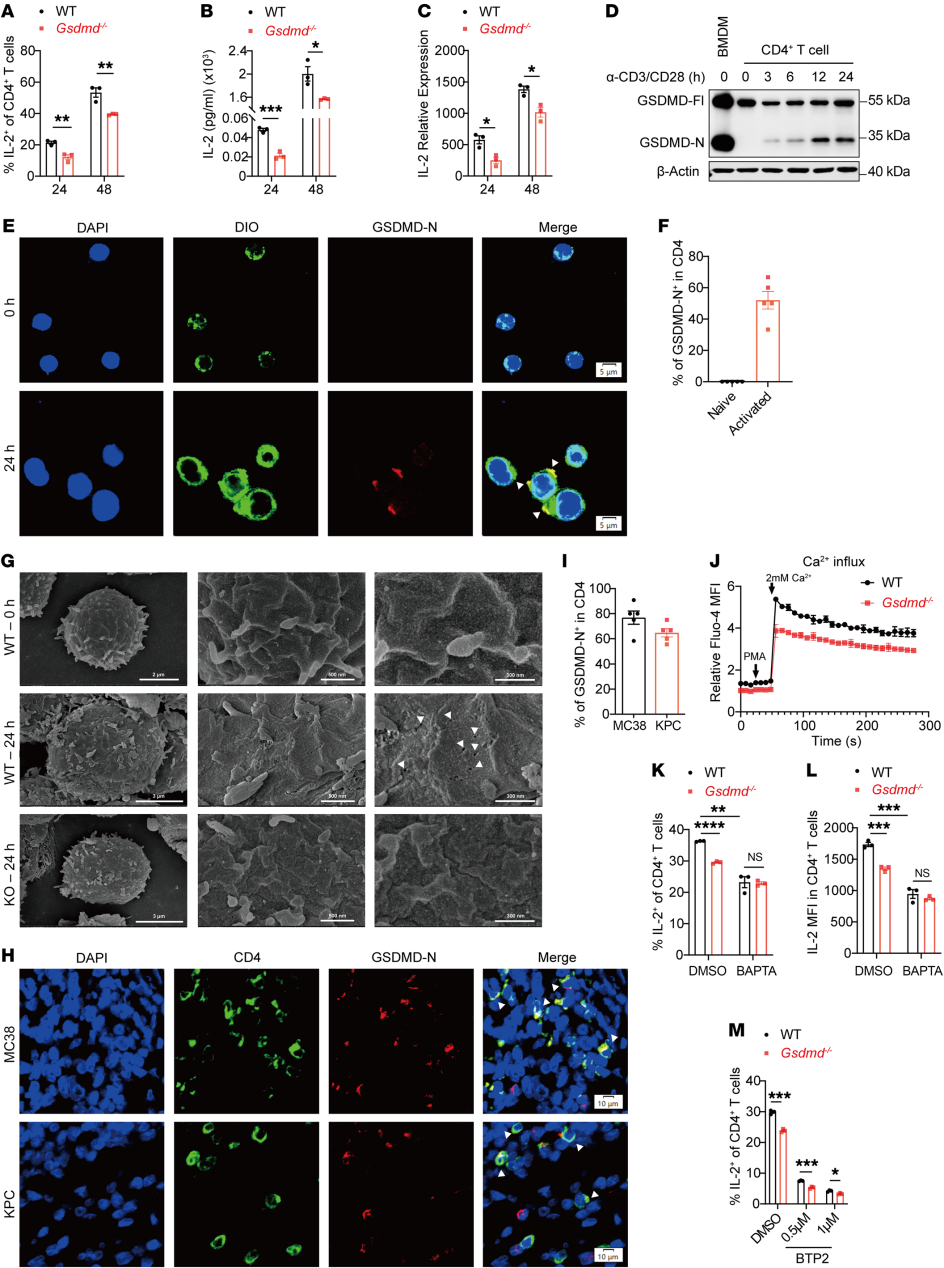
GSDMD-N pores mediate Ca^2+^ influx for induction of IL-2 in CD4^+^ T cells. (**A**–**C**) Flow cytometry (**A**), ELISA quantification (**B**) and RT-qPCR analysis (**C**) of IL-2 expression by WT and *Gsdmd^-/-^* CD4^+^ T cells activated in vitro. (**D**) Immunoblot analysis of GSDMD activation in activated CD4^+^ T cells. LPS- and nigericin-treated bone marrow-derived macrophages (BMDMs) were used as the positive control of GSDMD activation. (**E** and **F**) Immunofluorescence staining for GSDMD-N and DiO (a cell membrane fluorescent probe) in activated human CD4^+^ T cells (**E**). The percentage of GSDMD-N^+^ cells among DiO^+^ cells were quantified from five fields of view (**F**). Scale bars: 5 µm. (**G**) Scanning electron microscope (SEM) analysis of membrane pores in naive WT CD4^+^ T cells and TCR-activated WT or *Gsdmd^–/–^* CD4^+^ T cells. The white arrowheads indicate the membrane pore formation in CD4^+^ T cells. Scale bars: 2 µm (WT, 0 h; left); 3 µm (WT and KO, 24 h; left); 300 nm (WT, 0 h and 24 h, and KO 24 h; right); 500 nm (WT, 0 h and 24 h, and KO 24 h; middle). (**H** and **I)** Immunofluorescence staining for GSDMD-N and CD4 in MC38 (top) and KPC (bottom) tumor tissues (**H**). The percentages of GSDMD-N^+^ cells among CD4^+^ cells (**I**). Scale bars: 10 µm. The white arrowheads indicate GSDMD-N and CD4 co-expressing cells. (**J**) Time-course analysis of Ca^2+^ influx in activated WT and *Gsdmd^–/–^* CD4^+^ T cells in response to PMA stimulation. (**K**–**M**) Percentages of IL-2-expressing CD4^+^ T cells (**K**) and IL-2 MFI in WT and *Gsdmd^-/-^* CD4^+^ T cells (**L** and **M**) activated in vitro in the presence or absence of BAPTA (50 µM) (**L**) or BTP2 (**M**). Data are presented as mean ± SEM (**A**–**C**, **I**–**M**, *n* = 3 per group) and are representative of at least two independent experiments (**A**–**M**). *P < 0.05; **P < 0.01; ***P < 0.001; ****P < 0.0001; NS, not significant; as determined by unpaired 2-tailed Student’s *t* tests.

We next investigated the mechanism by which GSDMD induced IL-2 production in CD4^+^ T cells. Flow cytometry analysis of in vitro–activated CD4^+^ T cells showed that *Gsdmd^–/–^* CD4^+^ T cells expressed less IL-2 than WT counterparts at 24 and 48 hours after activation (Figure 5A), which was further confirmed by measurement of the secreted IL-2 concentrations (Figure 5B). These protein alterations were associated with changes in IL-2 transcript levels (Figure 5C), suggesting that GSDMD may regulate IL-2 transcription in response to TCR activation. Further, inactivation of GSDMD by DSF treatment inhibited IL-2 expression similarly to GSDMD depletion (Supplemental Figure 5, A–C). To investigate the effect of DSF on IL-2 expression by CD4^+^ T cells in response to cognate antigen encounter, we activated OT-II cells with OVA_323–339_ peptide in the presence or absence of DSF. As expected, DSF treatment consistently led to reduced IL-2 expression at both protein and transcript levels (Supplemental Figure 5, D–F). These data support a crucial role of GSDMD in IL-2 transcription in activated CD4^+^ T cells.

GSDMD proteins form pores after cleavage to execute biological functions. Indeed, we found that GSDMD was cleaved to generate an active GSDMD-N fragment rapidly after TCR stimulation of CD4^+^ T cells (Figure 5D). Immunofluorescence staining with the plasma membrane marker DiO and anti–GSDMD-N showed that the cleaved GSDMD-N localized on the plasma membrane of activated CD4^+^ T cells but not naive CD4^+^ T cells (Figure 5, E and F), indicating the pore-forming activity of GSDMD-N. Indeed, we observed pores in the plasma membrane of CD4^+^ T cells, formed with GSDMD presence and TCR stimulation, by scanning electron microscopy (Figure 5G). Moreover, GSDMD-N accumulation was also found on the plasma membrane of tumor-infiltrating CD4^+^ T cells in tumor sections (Figure 5, H and I). Thus, GSDMD is cleaved to form plasma membrane pores in activated CD4^+^ T cells.

Cleaved GSDMD-N forms pores in the cell membrane that typically cause cell pyroptosis (30). However, in activated CD4^+^ T cells, lactate dehydrogenase (LDH) release was not affected by GSDMD depletion (Supplemental Figure 5G). Cell death level reflected by the percentage of prodium iodide (PI^hi^) cells was not changed in the *Gsdmd^–/–^* CD4^+^ T cells either (Supplemental Figure 5H). In addition, we did not observe the characteristic manifestation of pyroptosis, i.e., cell swelling and membrane rupture, in CD4^+^ T cells following TCR activation (Supplemental Figure 5I and Supplemental Video 1 [Activated WT CD4 T cells] and Supplemental Video 2 [Activated KO CD4 T cells]These data thus suggest a nonpyroptotic function of GSDMD activation in CD4^+^ T cells.

Besides pyroptosis, GSDMD-formed pores have recently been reported to mediate ion flux, including K^+^ efflux and Ca^2+^ influx (31–33). Given the fact that Ca^2+^ influx following TCR activation is a pivotal event in activating NFATs responsible for IL-2 transcriptional activation (34), we speculated that IL-2 transcription induced by GSDMD activation in CD4^+^ T cells was possibly linked to Ca^2+^ influx. To test this, we performed the calcium flux assay and observed that *Gsdmd^–/–^* CD4^+^ T cells showed an intracellular Ca^2+^ level comparable to that of WT cells at the beginning of stimulation, but tended to exhibit a lower Ca^2+^ level at 10 minutes of anti-CD3/CD28 stimulation (Supplemental Figure 5J), coinciding with the occurrence of GSDMD cleavage (Supplemental Figure 5K). In line with the intracellular Ca^2+^ dynamics, NFAT dephosphorylation was reduced in the *Gsdmd^–/–^* CD4^+^ T cells at 10 minutes after TCR activation when GSDMD activation was evident (Supplemental Figure 5K). Further, in preactivated CD4^+^ T cells, GSDMD deficiency or DSF treatment reduced the immediate Ca^2+^ influx in response to PMA treatment as a result of the lack of GSDMD-N pores (Figure 5J and Supplemental Figure 5L).

When BAPTA was used to chelate extracellular Ca^2+^, the level of IL-2 secreted by CD4^+^ T cells was significantly decreased, whereby the reduction in IL-2 expression caused by GSDMD knockout was no longer observed (Figure 5, K and L). As Ca^2+^ release–activated Ca^2+^ (CRAC) channels are the predominant Ca^2+^ influx pathway in lymphocytes (35), BTP2, a selective blocker of CRAC channels, expectedly suppressed IL-2 production, but it did not interfere with the suppressive effect of GSDMD deficiency on IL-2 production in CD4^+^ T cells (Figure 5M), indicating that GSDMD-N pores mediate Ca^2+^ influx independent of the classical calcium ion channels. Collectively, these data support a role of GSDMD-N pores in mediating Ca^2+^ influx upon TCR activation, which is required for optimal IL-2 production by CD4^+^ T cells.

This newly identified function of GSDMD seemed to be specific to CD4^+^ T cells, since we could not detect any cleaved GSDMD in activated CD8^+^ T cells by Western blot (Supplemental Figure 5M). Correspondingly, GSDMD-deficient CD8^+^ T cells displayed no defects in IL-2 production (Supplemental Figure 5, N and O), consistent with the in vivo results (Figure 4, F–H). The biological role of GSDMD in CD8^+^ T cells remains elusive and is worthy of future investigation.

### Caspase-8 activation mediates GSDMD cleavage upon TCR activation, susceptible to suppression by tumor-derived factors.

**Figure 6:**
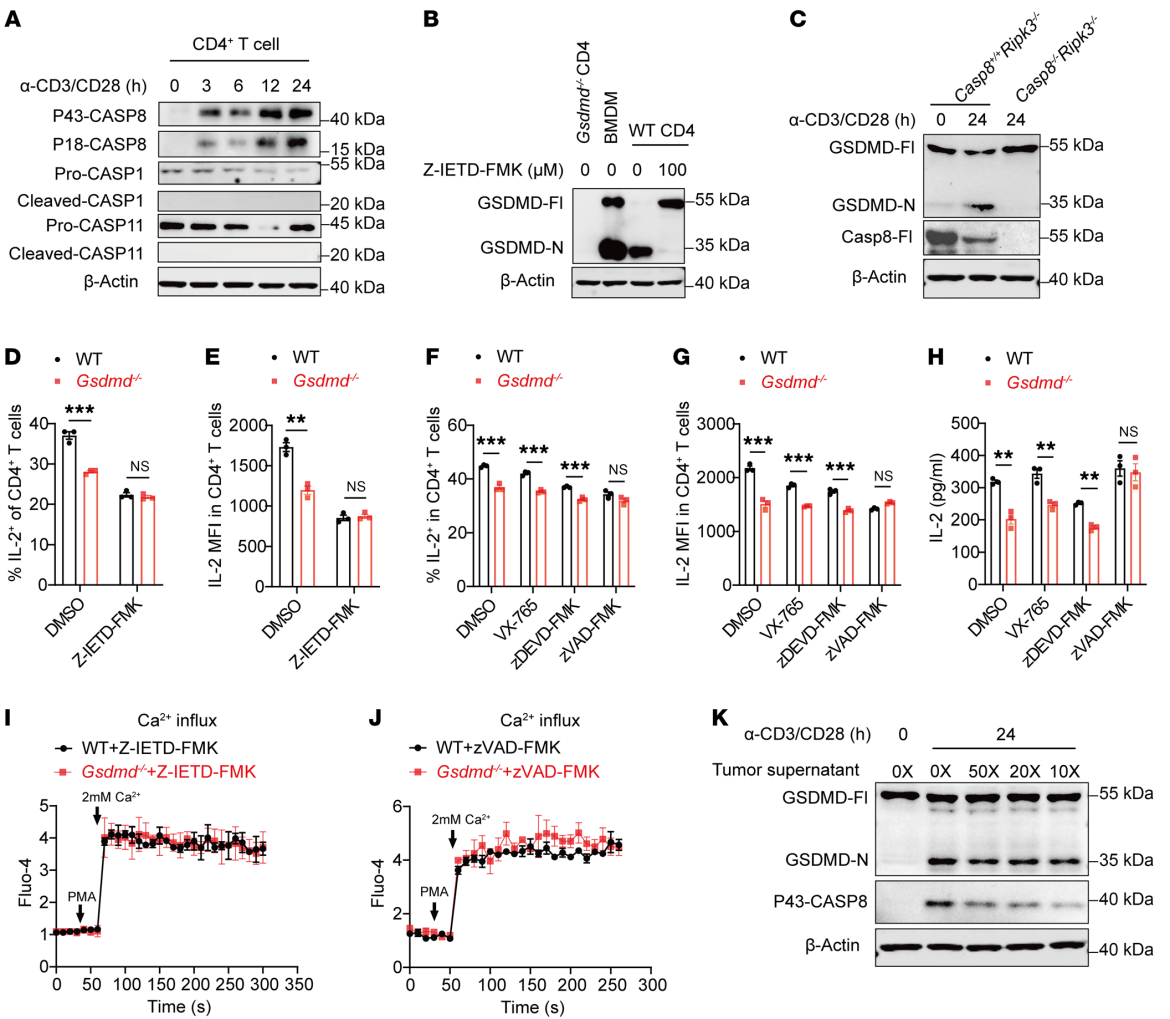
Caspase-8 mediates GSDMD cleavage in CD4^+^ T cells after TCR activation, and GSDMD is suppressed by tumor cell–derived proteins. (**A**) Immunoblot analysis of caspase cleavage in CD4^+^ T cells activated in vitro by anti-CD3/CD28 for the indicated times. (**B**) Immunoblot analysis of GSDMD activation in CD4^+^ T cells activated in vitro by anti-CD3/CD28 for 24 hours with or without the addition of a caspase-8 inhibitor (Z-IETD-FMK, 100 μM). (**C**) Immunoblot analysis of GSDMD activation in CD4^+^ T cells isolated from *Caspase-8^+/+^ Ripk3^–/–^* and *Caspase-8^–/–^ Ripk3^–/–^* mice and activated in vitro by anti-CD3/CD28 for 24 hours. (**D** and **E**) Percentages of IL-2–expressing CD4^+^ T cells (**D**) and IL-2 MFI in CD4^+^ T cells (**E**) isolated from WT and *Gsdmd^–/–^* mice and activated in vitro in the presence or absence of Z-IETD-FMK for 24 hours. (**F**–**H**) WT and *Gsdmd^-/-^* CD4^+^ were treated with or without caspase-1 inhibitor (VX-765, 20 µM), caspase-3 inhibitor (zDEVD-FMK, 20 µM) or pan-caspase inhibitor (zVAD-FMK, 20 µM) during in vitro activation by anti-CD3/CD28 for 24 hours. Percentages of IL-2-expressing CD4^+^ T cells (**F**) and IL-2 MFI in CD4^+^ T cells (**G**) were analyzed. Secreted IL-2 was quantified by ELISA (**H**). (**I** and **J**) Time course analysis of Ca^2+^ influx in WT and *Gsdmd^–/–^* CD4^+^ T cells activated in vitro with or without the addition of Z-IETD-FMK (**I**) or zVAD-FMK (**J**). (**K**) Immunoblot analysis of GSDMD and caspase-8 protein activation in CD4^+^ T cells activated in vitro by anti-CD3/CD28 for 24 hours with or without MC38 tumor supernatant at different dilutions. Data are presented as mean ± SEM (**D**–**J**, *n* = 3) and are representative of at least 2 independent experiments. **P < 0.01, ***P < 0.001, NS, not significant, as determined by unpaired 2-tailed Student’s *t* tests.

Several caspases, including caspase-1, -8, and -11, have been reported to cleave GSDMD (30, 36, 37). We then investigated which caspase is responsible for GSDMD cleavage in activated CD4^+^ T cells. We found that caspase-8, but not caspase-1 or -11, was activated in CD4^+^ T cells after anti-CD3/CD28 stimulation (Figure 6A), which was consistent with a previous report that caspase-8 undergoes limited activation after antigenic stimulation in human CD4^+^ T cells (38). Targeting caspase-8 with an inhibitor, Z-IETD-FMK, or by genetic depletion was sufficient to abolish the TCR stimulation–induced GSDMD activation in CD4^+^ T cells (Figure 6, B and C), while in contrast the GSDMD cleavage was still observed in the *Casp1^–/–^* or *Casp11^–/–^* CD4^+^ T cells (Supplemental Figure 6, A and B). Notably, *Ripk3^–/–^ Casp8^–/–^* mice were used for isolating CD4^+^ T cells with *Ripk3^–/–^* mice as control, because of embryonic lethality of *Casp8^–/–^* mice (39) (Figure 6C). Correspondingly, treatment with a caspase-8 inhibitor significantly reduced IL-2 production by CD4^+^ T cells, whereby the effect of GSDMD depletion on IL-2 production was no longer manifested (Figure 6, D and E). In contrast, inhibiting caspase-1 or caspase-3 with VX-765 and zDEVD-FMK, respectively, did not alter GSDMD depletion’s effect on IL-2 production (Figure 6, F–H). Further, the difference in Ca^2+^ influx between WT and *Gsdmd^–/–^* CD4^+^ T cells was diminished by caspase-8 inhibition (Figure 6I) and pan-caspase inhibition (Figure 6J), but not by inhibition of caspase-1 or caspase-3 (Supplemental Figure 6, C and D). These data identify caspase-8 as the enzyme responsible for GSDMD cleavage in CD4^+^ T cells in response to TCR activation. Interestingly, caspase-8 was not activated in CD8^+^ T cells after anti-CD3/CD28 stimulation (Supplemental Figure 5M), which could explain the absence of GSDMD cleavage in CD8^+^ T cells.

Intratumoral T cells often become dysfunctional in the TME as a result of diverse immunosuppressive factors, including cytokines, metabolites, ions, and other components (40, 41). It is possible that the limited IL-2 production by intratumoral CD4^+^ T cells could be in part due to GSDMD inactivation, since a portion of CD4^+^ T cells did not display GSDMD activation in tumor sections. To test this idea, we treated CD4^+^ T cells with tumor tissue supernatant during their in vitro activation, and found that cleavage of GSDMD and of caspase-8 were both weakened (Figure 6K), leading to reduced production of IL-2 (Supplemental Figure 6, E and F). Consistently, conditioned medium from cultured cells, including MC38, KPC, and B16-OVA, noticeably reduced the activation of caspase-8 and GSDMD in CD4^+^ T cells (Supplemental Figure 6, G and H), an effect no longer observed when conditioned medium was heat-denatured (Supplemental Figure 6I). These results implicate that certain tumor cell–derived factors, possibly proteins, may target GSDMD in CD4^+^ T cells to facilitate immune evasion.

### GSDMD activation in intratumoral CD4^+^ T cells is associated with favorable prognosis and improved response to anti–PD-1 immunotherapy in human cancers.

**Figure 7:**
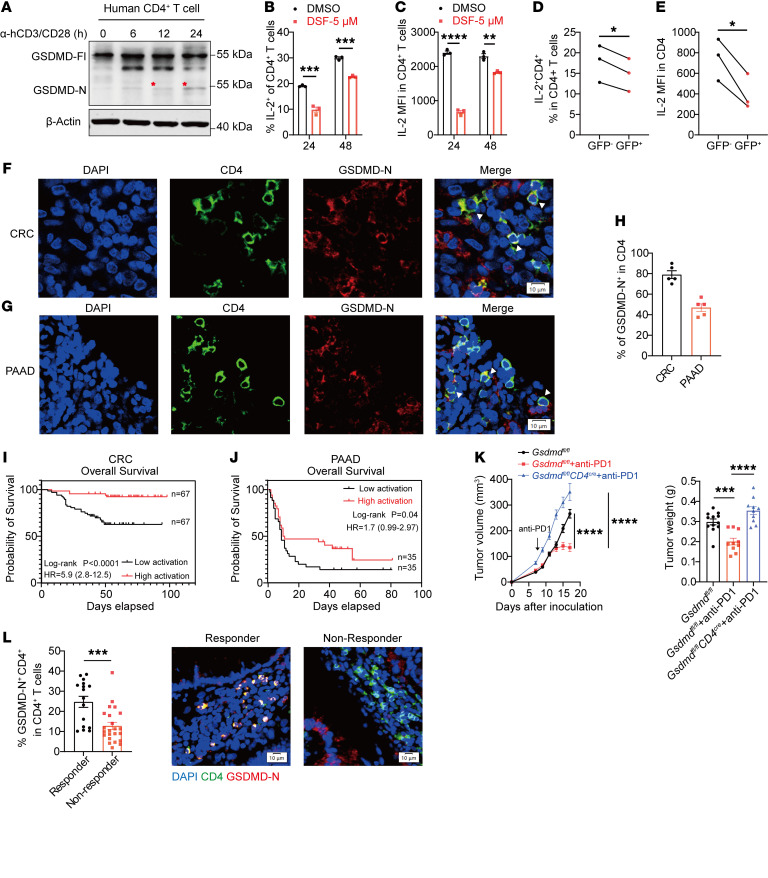
GSDMD activation in CD4^+^ T cells correlates with survival benefit and immunotherapy efficacy in colorectal cancer and pancreatic adenocarcinoma. (**A**) Immunoblot analysis of GSDMD protein activation probed with a mixture of full-length and N-fragment–specific GSDMD antibodies in human CD4^+^ T cells isolated from PBMCs and activated by anti-hCD3/hCD28 for the indicated times. Red asterisks indicate cleaved GSDMD fragments. (**B** and **C**) Flow cytometry analysis of percentages of IL-2–expressing human CD4^+^ T cells (**B**) and IL-2 MFI in human CD4^+^ T cells (**C**) activated in vitro for 24 hours in the presence or absence of DSF (*n* = 3 per group). (**D** and **E**) Percentages of IL-2–expressing cells within shGSDMD-GFP–transduced (GFP^+^) or nontransduced (GFP^–^) human CD4^+^ T cells (**D**) and IL-2 MFI of GFP^–^ and GFP^+^ human CD4^+^ T cells (**E**). (**F**–**H**) Immunofluorescence staining for GSDMD-N (red) and CD4 (green) in tumor tissues from patients with colorectal cancer (**F**) or pancreatic cancer (**G**). Percentages of GSDMD-N^+^ cells among CD4^+^ cells were quantified from 5 independent fields of view within colorectal cancer (CRC) and pancreatic adenocarcinoma (PAAD) (**H**). Scale bars: 10 μm. White arrowheads indicate GSDMD-N– and CD4-coexpressing cells. (**I** and **J**) Patients with colorectal cancer (**I**) or pancreatic cancer (**J**) were stratified into high-activation and low-activation groups based on the median ratio of GSDMD-active CD4^+^ TILs to total CD4^+^ TILs, and Kaplan-Meier curves of overall survival were calculated. (**K**) Tumor growth curves (left) and tumor weights (right) of MC38 tumors implanted in *Gsdmd^fl/fl^* and *Gsdmd^fl/fl^ CD4^cre^* mice treated with anti–PD-1. (**L**) Left: Percentages of GSDMD-N^+^ cells in tumor-infiltrating CD4^+^ T cells in immunotherapy-responsive and –nonresponsive colorectal cancer patients. Right: Representative images of GSDMD activation in tumor-infiltrating CD4^+^ T cells. Data are presented as mean ± SEM (**B**–**E** and **H**–**L**) and are representative of at least 2 independent experiments (**B**–**H** and **K**). *P < 0.05, **P < 0.01, ***P < 0.001, ****P < 0.0001, as determined by unpaired 2-tailed Student’s *t* tests (**B**, **C**, and **L**), paired 2-tailed Student’s *t* test (**D** and **E**), 1-way ANOVA for tumor weight (**K**), or log-rank test (**I** and **J**).

We next sought to validate these findings in human cells. Isolated CD4^+^ T cells from donors’ peripheral blood also displayed cleaved GSDMD upon in vitro stimulation (Figure 7A). IL-2 production by activated CD4^+^ T cells was significantly reduced by DSF treatment (Figure 7, B and C). In addition, we performed shRNA-mediated GSDMD knockdown in human CD4^+^ T cells and also detected a decreased IL-2 level in GSDMD-knockdown cells (GFP^+^) compared with control T cells (GFP^–^) (Figure 7, D and E). Using confocal microscopy to interrogate tumor tissues, GSDMD activation in CD4^+^ T cells within both human colonic and pancreatic tumor tissues was further confirmed by anti–GSDMD-N staining (Figure 7, F–H). These results demonstrate GSDMD activation in human intratumoral CD4^+^ T cells.

We next assessed correlations between GSDMD expression and tumor immune infiltration in human cancers by analyzing the TISIDB and TIMER databases. In various types of cancer, GSDMD expression was positively correlated with active CD8^+^ T cells (Supplemental Figure 7A). In colon adenocarcinoma (COAD) and pancreatic adenocarcinoma (PAAD) patients, GSDMD expression was also associated with Th1 infiltration (Supplemental Figure 7B), characterized by IFN-γ secretion.

Based on the earlier demonstration that GSDMD activation in CD4^+^ T cells is critical for antitumor immunity in animal models, we further examined the correlation between tumor prognosis and GSDMD activation levels in tumor-infiltrating CD4^+^ T cells by conducting tissue microarray–based immunofluorescence in a single-center retrospective cohort study. Ratios of GSDMD-active over total CD4^+^ T cells were calculated. The median ratio was set as the cutoff value to divide patients into high- and low-activation groups (Supplemental Figure 7C). By integrating the prognostic information of patients, we found that high activation of GSDMD in the intratumoral CD4^+^ T cells was associated with survival benefit in both COAD and PAAD patients (Figure 7, I and J). These data indicate that GSDMD activation may also be required for human CD4^+^ T cells to potentiate antitumor immunity.

PD-1 blockade in combination with IL-2 administration is an emerging approach in treating cancer patients (42, 43), in which IL-2 improves PD-1 blockade therapy through skewing the differentiation program of PD-1^+^TCF1^+^ stem-like CD8^+^ T cells away from T cell exhaustion, while instead toward effector T cells (44). In the subcutaneous MC38 tumor model, anti–PD-1 treatment caused tumor regression, while progressive tumor expansion was observed in the *Gsdmd^fl/fl^ CD4^cre^* mice after anti–PD-1 treatment, indicating the importance of GSDMD activation in CD4^+^ T cells for the therapeutic effectiveness (Figure 7K). Based on this finding, we further examined GSDMD activation in tumor-infiltrating CD4^+^ T cells of colorectal tumor tissues derived from patients responsive and patients nonresponsive to anti–PD-1 immunotherapy. After detection of tumor-infiltrating CD4^+^ T cells and GSDMD-N expression by immunofluorescence, the ratio of GSDMD-N^+^ CD4^+^ T cells was calculated. The results showed that patients responding to anti–PD-1 immunotherapy had a higher activation ratio of GSDMD in CD4^+^ T cells than nonresponders (Figure 7L), indicating a positive correlation of GSDMD activation in CD4^+^ T cells and immunotherapy efficacy.

## Discussion

Pyroptosis of tumor cells mediated by gasdermin family proteins can provoke antitumor immune response and has emerged as a potential target for cancer immunotherapy (18, 19). However, whether and how gasdermins intrinsically regulate immune cells in tumor immunity remains unclear. In this study, we demonstrated, for the first time to our knowledge, that GSDMD in T lymphocytes is required for antitumor immunity. Both genetic and chemical approaches in mouse models revealed an essential role of GSDMD in antitumor immunity, which relied on its activity in CD4^+^ T lymphocytes and its inducing effect on IL-2 production. CD8^+^ T cells are prominent effector cells in antitumor immunity, but they have relatively weak ability to secrete IL-2, so their activation and proliferation often require the help of CD4^+^ T cells (7). Significantly, this study identified that GSDMD activation in CD4^+^ T cells is critically involved in this process.

Several recent studies have reported new functions of GSDMD pores in the plasma membrane besides induction of pyroptosis (31, 33, 45). For example, Zhang et al. reported that the activation of GSDMD in intestinal epithelial cells drove mucin secretion through Ca^2+^-dependent, scinderin-mediated cortical F-actin disassembly (33). We found that GSDMD activation in activated CD4^+^ T cells promoted IL-2 expression by enhancing Ca^2+^ influx without inducing pyroptosis. We did not observe any typical pyroptotic characteristics in activated CD4^+^ T cells, which is consistent with a previous finding that CARD8/caspase-1–mediated pyroptosis cannot be engaged in activated human CD4^+^ T cells (46). Similarly, in another study, human Th17 cells were found to be resilient to pyroptosis despite GSDME-formed pores in the cell membrane after TCR stimulation (47). Though both CARD8/caspase-1 activation and TCR signaling can induce GSDMD cleavage in human CD4^+^ T cells, CARD8/caspase-1 activation triggered a stronger GSDMD activation (data not shown). We have compared the number of pores between TCR-activated CD4^+^ T cells and macrophages treated with LPS/ATP, known to induce GSDMD-dependent pyroptosis. LPS/ATP–treated macrophages display many more pores in the cell membrane than activated CD4^+^ T cells (data not shown). It is possible that the limited formation of pores in activated CD4^+^ T cell membrane might act below the threshold of triggering pyroptosis. Further studies are needed to elucidate the underlying mechanism by which these specialized cells can evade GSDMD-mediated pyroptosis.

We showed that GSDMD was required for the effector function of CD4^+^ T cells, which typically relies on cytokine production. Accelerated tumor growth in the *Gsdmd^fl/fl^ CD4^cre^* mice was associated with decreased cytokine levels, including IL-2, IL-12, IL-4, and IL-10. IL-12 is a cytokine primarily produced by DCs and monocytes/macrophages, and promotes Th1 differentiation. Production of bioactive IL-12 in those cells can be amplified by activated T cell–derived signals, for instance IFN (48) and CD40L (5). Thus, the decreased level of IL-12 is likely a consequence of the impaired T cell function in the absence of GSDMD. IL-10 is generally considered as an immunosuppressive cytokine in the TME (49), so the decreased level of IL-10 is unlikely to be the reason for the accelerated tumor outgrowth, while the role of IL-4 in antitumor immunity is ambiguous (50). The help from CD4^+^ T cell–derived IL-2 in the tumor milieu is required for the full response of CD8^+^ cytotoxic T cells (7, 14). Therefore, we proposed IL-2 as the major effector cytokine regulated by GSDMD in CD4^+^ T cells, which was then consolidated by multiple lines of evidence. IL-2 not only promotes the survival and expansion of effector T cells and NK cells, but also facilitates the development and maintenance of immunosuppressive CD4^+^Foxp3^+^ Tregs (51). In this study, we also observed a significant reduction in tumor-infiltrating Tregs (CD4^+^Foxp3^+^ T cells) in GSDMD-deficient or DSF-treated mice, presumably due to IL-2 reduction (data not shown).

Several caspases can cleave GSDMD to generate a GSDMD-N terminus with pore-forming activity under certain pathological conditions (52). Antigen stimulation has been previously reported to induce limited caspase-8 activation in human CD4^+^ T cells, generating proliferative signaling while avoiding apoptosis (38). Consistently, we observed that TCR stimulation induced caspase-8 activation in mouse CD4^+^ T cells as early as 3 hours. Caspase-8 inhibitor not only abolished the TCR signaling–induced GSDMD cleavage but also diminished the effect of GSDMD loss on Ca^2+^ influx and IL-2 secretion. Thus, our study uncovered what we believe to be a new mechanism by which caspase-8 promotes T cell proliferation, i.e., cleaving GSDMD to form GSDMD pores that allow calcium influx to trigger IL-2 production. Notably, GSDMD had no effect on IL-2 production in CD8^+^ T cells, likely because the cleavage of caspase-8 and GSDMD did not occur in activated CD8^+^ T cells, but the underlying mechanism needs to be further explored.

Disulfiram (DSF), an old FDA-approved drug for alcohol dependence treatment, has been reported to affect tumor growth in several ways (53–55). DSF-mediated direct tumor-killing activity has been reported in certain tumor cell lines and is known to be associated with mechanisms linked to NPL4 (53). Terashima et al. found that DSF targeted FROUNT (also known as nucleoporin 85) to suppress TAM accumulation and inhibited tumor growth (54). A recent study showed that DSF directly activated LCK-mediated TCR signaling in CD8^+^ T cells to promote antitumor immunity (56). Oppositely, an immunosuppressive effect of DSF on T cell infiltration was also reported, in which it upregulated PD-L1 expression in hepatocellular carcinoma cells to suppress T cell infiltration (57). In this study, we demonstrated that DSF, as an inhibitor of GSDMD-N oligomerization, promoted tumor growth by suppressing CD4^+^ T cell–mediated antitumor immunity. DSF had no effect on viability of MC38 cells used in our study (data not shown). In addition, we did not observe any difference in the intratumoral accumulation of macrophages between DSF-treated and control tumors (Supplemental Figure 2, B and K). Intriguingly, DSF treatment impaired LCK activation in both tumor-infiltrating CD4^+^ and CD8^+^ T cells (data not shown). Though Wang et al. identified that DSF promotes TCR signaling–triggered LCK activation in CD8^+^ T cells in vitro, they did not examine the impact of DSF on LCK activation in vivo (56). These findings underscore that the effects of DSF on tumor progression are multifaceted, which may depend on the tumor models, treatment regimens, and so on. An increasing number of clinical trials have been enrolled to evaluate the antitumor efficacy of DSF in solid malignancies (58). However, DSF only shows limited efficacy thus far, and, for instance, it appears to only prolong survival in patients with newly diagnosed non–small cell lung cancer (58). The clinical study of DSF for colorectal cancer and pancreatic adenocarcinoma patients has not been reported yet. Given the complicated effect of DSF on antitumor immunity, more caution and careful design are warranted in clinical trials of DSF.

Gasdermin-mediated pyroptosis directly kills tumor cells and provokes a robust antitumor immune response. In this study, we uncovered a CD4^+^ T cell–intrinsic role of GSDMD in potentiating antitumor immunity independent of the canonical pyroptosis. This finding is particularly clinically relevant as GSDMD activation in CD4^+^ T cells was positively correlated with patients’ prognosis and immunotherapy response. Considering the finding that tumor cell–derived factors suppressed caspase-8 and GSDMD activation in CD4^+^ T cells, it will be intriguing to identify and characterize these factors in the TME, which could be potential new targets for cancer immunotherapy.

## Methods

### Sex as a biological variable.

Our study used biopsies from both male and female humans and mice, as sex was not considered as a biological variable.

### Mice.

Six- to eight-week-old C57BL/6J mice were purchased from the Model Animal Research Center of Nanjing University (Nanjing, China). *Gsdmd^–/–^* mice were gifts from Feng Shao at the National Institute of Biological Science, Beijing, China. *Gsdmd^fl/fl^* mice were purchased from GemPharmatech. *Rag2^–/–^*, CD45.1, and OT-II mice were gifts from Zhijian Cai at Zhejiang University School of Medicine. *Gsdmd^fl/fl^* mice were crossed with CD4^cre^, Vav^cre^, or Lysm^cre^ mice to obtain *Gsdmd^fl/fl^ CD4^cre^*, *Gsdmd^fl/fl^ Vav^cre^*, and *Gsdmd^fl/fl^ Lysm^cre^* mice. Deletion efficiency was analyzed by immunoblotting. *Caspase-1^–/–^* and *Caspase-11^–/–^* mice were gifts from Ben Lv at Central South University Xiangya Hospital Changsha, China. *Ripk3^–/–^* and *Ripk3^–/–^ Casp8^–/–^* mice were provided by Jiahuai Han of Xiamen University, Xiamen, China. The mice were bred and maintained in a specific pathogen–free facility at the Laboratory Animal Center of Zhejiang University.

### Cell lines.

MC38 was purchased from ATCC. B16-OVA and KPC were provided by Yunhua Liu at Zhejiang University School of Medicine. *Gsdmd^–/–^* MC38 was generated by CRISPR/Cas9. Guide RNA oligos with sequences targeting the GSDMD genomic sequence (5′-ACCGCAGTAGGGCCTGAAGCTGG-3′ and 5′-AACCCAGCTTCAGGCCCTACTGC-3′) were cloned into the plasmid pEP-ko (pEP-330x). To generate GSDMD-knockout cells, these constructs were transfected into MC38 cells with Lipofectamine 3000 (Invitrogen). The cells were then seeded into 96-well plates to select single clones.

### Mouse tumor models.

MC38 (5 × 10^5^), KPC (1 × 10^6^), or B16-OVA (1 × 10^6^) cells in 100 μL PBS were injected subcutaneously on the right flank. Tumor measurements were performed using a caliper every 2–3 days. To test the effect of disulfiram (DSF) on antitumor immunity, control vehicle or DSF (40 mg/kg) was injected intraperitoneally (i.p.) daily from 1 day before tumor incubation. To test the effect of dimethyl fumarate (DMF) on antitumor immunity, control vehicle or DMF (100 mg/kg/d) was added into the water for mice. Mice received the water containing vehicle or DMF for 6 weeks. For the orthotopic KPC tumor model, 5 × 10^5^ KPC cells were injected into the pancreas via laparotomy. To test the role of IL-2 in GSDMD-knockout mice, tumor-bearing WT, *Gsdmd^–/–^*, *Gsdmd^fl/fl^*, or *Gsdmd^fl/fl^ CD4^cre^* mice were gavaged with FK506 (2 mg/kg) daily from day 4 after tumor incubation. For IL-2 treatment, a total of 1.5 μg/body mouse recombinant IL-2 (GenScript, Z02764) and 15 μg/body anti–IL-2 mAb (Bio X Cell, clone S4B6-1) were mixed and incubated at room temperature for 30 minutes. WT and *Gsdmd^–/–^* mice were injected with IL-2/S4B6-1 complexes i.p. every other day. Tumor volumes were determined by the formula 0.5 × *L* × *W*^2^, where *L* is the length and *W* is the width. Mice were euthanized before reaching humane endpoint or sacrificed at day 14–18 for tumor tissue harvesting.

### Immune cell or cytokine depletion.

For CD8^+^ and CD4^+^ T cell depletion, anti-CD8α depleting antibodies (Bio X Cell, clone YTS169.4) and anti-CD4 depleting antibodies (Bio X Cell, clone GK1.5) were injected i.p. into mice the day before tumor incubation at a dose of 200 μg, followed by injection of 100 μg every 3 days. For NK cell depletion, anti-NK1.1 depleting antibodies (clone PK136, made in-house) were injected i.p. into mice the day before tumor incubation at a dose of 200 μg, followed by injection of 100 μg every 2 days. For tumor-associated macrophage depletion, clodronate liposomes or control liposomes (Liposoma Technology) were injected intratumorally twice a week starting on day 3 after tumor implantation at a dose of 1 mg/mouse. For IL-18 and IL-1β depletion, anti–IL-18 (Bio X Cell, clone YIGIF74-1G7) and anti–IL-1β (Bio X Cell, clone B122) were injected i.p. into mice the day before tumor incubation at a dose of 200 μg, followed by injection of 100 μg three times a week.

### Isolation of TILs.

Tumors were harvested on day 14–18 after injection, minced with scalpels into fragments, and digested in RPMI 1640 containing 1 mg/mL collagenase IV (Worthington Biochemical Corp.) and 0.1 mg/mL DNase I (Solarbio) for 40–60 minutes in a shaker at 37°C. After digestion, the single-cell suspensions were obtained by filtering of the solution with a 70 μm cell strainer and centrifuged at 500*g* for 5 minutes at 4°C.

### Flow cytometry.

Surface marker staining was performed in FACS buffer containing 1× PBS supplemented with 1% FBS and 2 mM EDTA. For intracellular cytokine staining, cells were stimulated with phorbol 12-myristate 13-acetate (PMA) (50 ng/mL; MedChemExpress) and ionomycin (1 μg/mL; MedChemExpress) in the presence of brefeldin A (5 μg/mL; MedChemExpress) at 37°C for 4 hours, followed by staining of intracellular proteins using an Intracellular Fixation & Permeabilization Buffer Set kit (Thermo Fisher Scientific).

The following antibodies (BioLegend) were used in this study: anti–mouse CD45 (30-F11), CD4 (RM4-5), CD8 (53-6.7), B220 (RA3-6B2), CD3 (17A2), CD11b (M1/70), F4/80 (BM8), Ly6G (1A8), Ly6C (HK1.4), CD11c (N418), I-A/I-E (M5/114.15.2), NK1.1 (PK136), IL-2 (JES6-5H4), IFN-γ (XMG1.2), granzyme B (QA16A02), Ki67 (16A8), CD45.1 (A20), CD45.2 (104), CD44 (IM7), CD62L (MEL-14), CD69 (H1.2F3), and CD25 (PC61) and anti–human CD4 (RPA-T4) and IL-2 (MQ1-17H12).

### Adoptive transfer.

For adoptive transfer experiments, total CD4^+^ T cells and CD8^+^ T cells from WT, *Gsdmd^–/–^*, and CD45.1 mice were isolated using EasySep Mouse CD4^+^ T or CD8^+^ T Cell Isolation Kit (STEMCELL Technologies). A total of 3 × 10^6^ cells at a ratio of 2:1 (CD4/CD8) were mixed in 200 μL PBS and injected i.v. into *Rag^–/–^* mice.

### T cell activation and differentiation in vitro.

CD4^+^ and CD8^+^ T cells were isolated from spleens of WT and *Gsdmd^–/–^* mice. 5 × 10^5^ T cells were plated per well in a 24-well plate precoated with 1 μg/mL anti-CD3ε (145-2C11, BioLegend) in complete RPMI (RPMI 1640 supplemented with 10% FBS, 50 μM β-mercaptoethanol, 100 U/mL penicillin, 100 U/mL streptomycin, nonessential amino acids [Solarbio], sodium pyruvate [Solarbio], and HEPES buffer [Solarbio]) containing 0.5 μg/mL anti-CD28 (37.51, BioLegend). After activation, T cells were harvested for the following analysis at certain time points. For CFSE labeling, isolated T cells were stained with CellTrace CFSE (Invitrogen) for 30 minutes in a 37°C water bath in the dark before stimulation with anti-CD3/CD28 and IL-2 (10 ng/mL; PeproTech) in the culture medium. Flow cytometry was performed after 72 hours of stimulation. For T cell differentiation, naive CD4^+^ T cells were activated with 2 μg/mL plate-bound anti-CD3ε /CD28, and differentiated by the addition of the following cytokines for 3 days: 10 ng/mL IL-2 (PeproTech), 10 ng/mL IL-12 (PeproTech), and 10 μg/mL anti–IL-4 (11B11, Bio X Cell) for Th1 polarization; 10 ng/mL IL-2 (PeproTech), 10 ng/mL IL-4 (PeproTech), and 10 μg/mL anti–IFN-γ antibody (XMG1.2, Bio X Cell) for Th2 polarization; and 10 ng/mL IL-2 (PeproTech) and 3 ng/mL TGF-β (R&D Systems) for Treg polarization.

### Lentiviral transduction of T cells.

For GSDMD knockdown, the oligonucleotides with targeting sequences (shGSDMD, 5′-GGTTCTGCCCTCAACACTT-3′) were cloned into Lenti-X shRNA vector, and lentiviruses were packaged by transfection of 293T cells with 10 μg of shGSDMD plasmids together with 10 μg of psPAX2 and 5 μg of pMD2G. Human CD4^+^ T cells were sorted out from PBMCs using an EasySep Human CD4^+^ T Cell Isolation Kit (STEMCELL Technologies). For lentiviral transduction, human CD4^+^ T cells were plated in 6-well plates and stimulated by anti-hCD3/hCD28 for 24 hours. After stimulation, viral supernatant (1:1 vol/vol ratio) and 8 μg/mL Polybrene (Sigma-Aldrich) were added. Spinfection was performed at 32°C for 2 hours at 800*g*, and medium was changed after 2 hours. After resting for 48 hours, transduced human CD4^+^ T cells were cultured for another 24 hours with anti-hCD3/hCD28 stimulation and tested in functional assays.

### Ca^2+^ influx analysis.

After activation in vitro for 6 hours, CD4^+^ T cells were harvested in suspension, followed by labeling with 4 μg/mL Fluo-4 (Invitrogen) for 1 hour at 37°C. After washing with cold PBS, cells were labeled with fluorescence-conjugated anti-CD4 for 30 minutes on ice. The prepared cells were resuspended in Ca^2+^-free buffer and warmed for 20 minutes at room temperature, and then the baseline Fluo-4 MFI was detected by flow cytometry for 30 seconds, followed by stimulation with PMA immediately before flow cytometry analysis for another 30 seconds. Then, CaCl_2_ was added, and Fluo-4 MFI was detected for 5 or 10 minutes. Mean fluorescence ratios were plotted after analysis with FlowJo software (Tree Star).

### RNA extraction and quantitative PCR.

Total RNA was extracted using TRIzol reagent (Invitrogen). cDNA was synthesized using a Hifair II 1st Strand cDNA Synthesis Kit (Yeasen Biotechnology) according to the manufacturer’s instructions. Quantitative real-time PCR was performed with SYBR Green Master ROX (Vazyme) on a CFX Touch system (Bio-Rad). The relative mRNA level was calculated after normalizing to the expression of β-actin. The following primers were used: mβ-actin forward primer, 5′-AACAGTCCGCCTAGAAGCAC-3′; mβ-actin reverse primer, 5′-CGTTGACATCCGTAAAGACC-3′; mIL-2 forward primer, 5′-TGAGCAGGATGGAGAATTACAGG-3′; mIL-2 reverse primer, 5′-GTCCAAGTTCATCTTCTAGGCAC-3′.

### Immunofluorescence analysis.

Freshly isolated tumor tissues were fixed in periodate-lysine-paraformaldehyde (PLP) fixative at 4°C overnight, dehydrated, and mounted in paraffin following standard protocols. Paraffin-embedded tumor tissue sections were incubated with 5% bovine serum albumin plus 5% normal goat serum in PBS for 1 hour at 37°C for blocking, followed by incubation with primary antibodies at 4°C overnight and on the following day with secondary antibodies for 1 hour at room temperature. The following antibodies were used: rabbit anti-GSDMD (Abcam, ab210070; 1:200), rabbit anti–GSDMD-N (Abcam, ab215203; 1:200), and mouse anti–pan-cytokeratin (Abcam, ab7753; 1:200) for human tumor tissues; cleaved gasdermin D (Asp276) (E3E3P) rabbit mAb (Cell Signaling Technology, 10137; 1:300) for mouse tumor tissues; rat anti-CD4 (Abcam, ab288724; 1:200); and rat anti-CD8 (Abcam, ab316778; 1:200). Secondary antibodies were anti-rabbit goat antibodies conjugated with Alexa Fluor 594 (Proteintech, SA00013-4; 1:600) and anti-rat goat antibodies conjugated with Alexa Fluor 488 (EarthOx, E032240; 1:600). The images were taken with a Zeiss LSM 800 confocal microscope and processed using ImageJ (NIH) and Adobe Photoshop CS6 software.

### Scanning electron microscopy.

After treatment of WT and *Gsdmd^–/–^* CD4^+^ T cells with or without anti-CD3/CD28, cells were washed with 1× PBS and then fixed in 2.5% glutaraldehyde solution at room temperature for 1–2 hours, then transferred to 4°C for overnight. After removal of the fixation solution, the cells were rinsed 3 times for 10–15 minutes each time with phosphate buffer (0.1 M, pH 7.4). Then cells were fixed with 1% osmium solution for 2 hours, and rinsed 3 times with 1× PBS (0.1 M, pH 7.4) for 10–15 minutes each time. Cells were dehydrated in an ethanol gradient (50%, 70%, 90%, 100%), and then dried at the critical point, coated, and observed with a Nova Nano 450 (Thermo FEI) scanning electron microscope. Images were analyzed with xT microscope Control v6.3.4 build 3233 software.

### Immunoblotting.

CD4^+^ and CD8^+^ T cells were isolated from WT mice and activated in vitro, and human CD4^+^ T cells were isolated from PBMCs of donors and activated in vitro. Proteins were electrophoresed and transferred to PVDF membranes. After blocking with Tris-buffered saline/Tween-20 (TBST) containing 5% skim milk for 1 hour at room temperature, the membranes were probed with the indicated primary antibody at 4°C overnight. After being washed 3 times, the membranes were incubated with HRP-conjugated secondary antibodies at 1:5,000 dilution for 1 hour at room temperature. The immunoblots were detected after application of ECL. Western blot analysis was performed using antibodies against mouse GSDMD (Abcam, ab209845); human GSDMD (Abcam, ab210070) and GSDMD-N (Abcam, ab215203); GAPDH (Abcam, ab8245); cleaved caspase-8 (Cell Signaling Technology, 8592), caspase-8-fl (Cell Signaling Technology, 4927), caspase-1 (Adipogen, AG-20B-0042-C100), and caspase-11 (Novus, NB120-10454).

### ELISA.

To determine the levels of IL-2, IL-1β, and IL-18 protein in the tumor microenvironment, tumors collected on day 18 after implantation were homogenized by mechanic disruption with magnetic beads in PBS buffer with complete protease inhibitors. After centrifugation, the supernatant was collected and measured for levels of IL-2 (Invitrogen, 88-7024-88), IL-1β (Invitrogen, BMS6002), and IL-18 (Invitrogen, BMS618-3), according to the manufacturer’s instructions. To determine the levels of IL-2 in the T cell culture medium, the CD4^+^ and CD8^+^ T cell culture medium was collected after stimulation by anti-CD3/CD28 for 24 hours.

### Cell death assay.

Cell death was determined by the lactate dehydrogenase release assay using the Pierce LDH Cytotoxicity Assay Kit (Beyotime) according to the manufacturer’s instructions.

### Patient survival analysis.

Colorectal carcinoma tissue microarray and the related patient survival data came from Sir Run Run Shaw Hospital affiliated with Zhejiang University. Pancreatic cancer patient survival data and tissue microarray were obtained from Shanghai Outdo Biotech Co. Ltd. Tissue sections of colorectal cancer patients receiving anti–PD-1 immunotherapy were from The First Affiliated Hospital of Zhejiang University. For survival analysis, patients with colonic cancer were stratified into high-activation (*n* = 67) and low-activation (*n* = 67) groups based on the median ratio of GSDMD-active CD4^+^ T cells to total tumor-infiltrating CD4^+^ T cells (median ratio was 48.08%). Patients with pancreatic cancer were stratified into high-activation (*n* = 35) and low-activation (*n* = 35) groups based on the median ratio of GSDMD-active CD4^+^ T cells to total tumor-infiltrating CD4^+^ T cells (median ratio was 2.55%). Standard Kaplan-Meier survival analysis and log-rank tests were used to determine the association of these groups with survival rate and the statistical significance.

### Live-cell imaging.

CD4^+^ T cells were isolated from WT or *Gsdmd^–/–^* mice followed by activation with anti-CD3/CD28 for 24 hours in vitro. After activation, morphology of CD4^+^ T cells was monitored continuously under a live-cell imaging microscope for 2 hours.

### Statistics.

Statistical analyses were performed using GraphPad Prism 8 software. Differences were considered significant at a *P* value less than 0.05. Unpaired 2-tailed Student’s *t* test was used to calculate the *P* values for comparisons of tumor weights, tumor-infiltrating cells, cytokine levels, and relative mRNA expression levels. Two-way ANOVA was used for multiple comparisons in tumor growth. Correlation studies were analyzed using the Pearson’s correlation factor *r*. Kaplan-Meier survival analysis was performed with the log-rank (Mantel-Cox) test.

### Study approval.

Our study was exempted from written informed consent because of its retrospective nature, and clinical data were retrieved from patients’ medical records under supervision by the Research Ethics Committee of The First Affiliated Hospital, Zhejiang University School of Medicine (IIT20210176B). All animal research was performed under a protocol approved by the Medical Experimental Animal Care Commission of Zhejiang University (authorized N.O. ZJU2015-040-01).

### Data availability.

The values for all data points in the graphs are reported in the Supporting Data Values file.

## Author contributions

YY, XW, and WS conceived and designed the study. YY, LW, NW, ML, and WL performed experiments. WJ and TZ provided the clinical samples. YY, XW, and WS analyzed the data and wrote the paper.

## Supplementary Material

Supplemental data

Unedited blot and gel images

Supplemental video 1

Supplemental video 2

Supporting data values

## Version 1 08/01/2025

Electronic publication
