# Supplementary material for: A CD4+ T lymphocyte–specific TCR/GSDMD/IL-2 axis facilitates antitumor immunity
Source: J Clin Invest. 2025 Aug 1;135(15):e191119. doi: 10.1172/JCI191119 (PMC12321394; doi:10.1172/JCI191119)
Supplement: Supplemental data [file jci-135-191119-s178.pdf]

Supplemental Figures and Legends

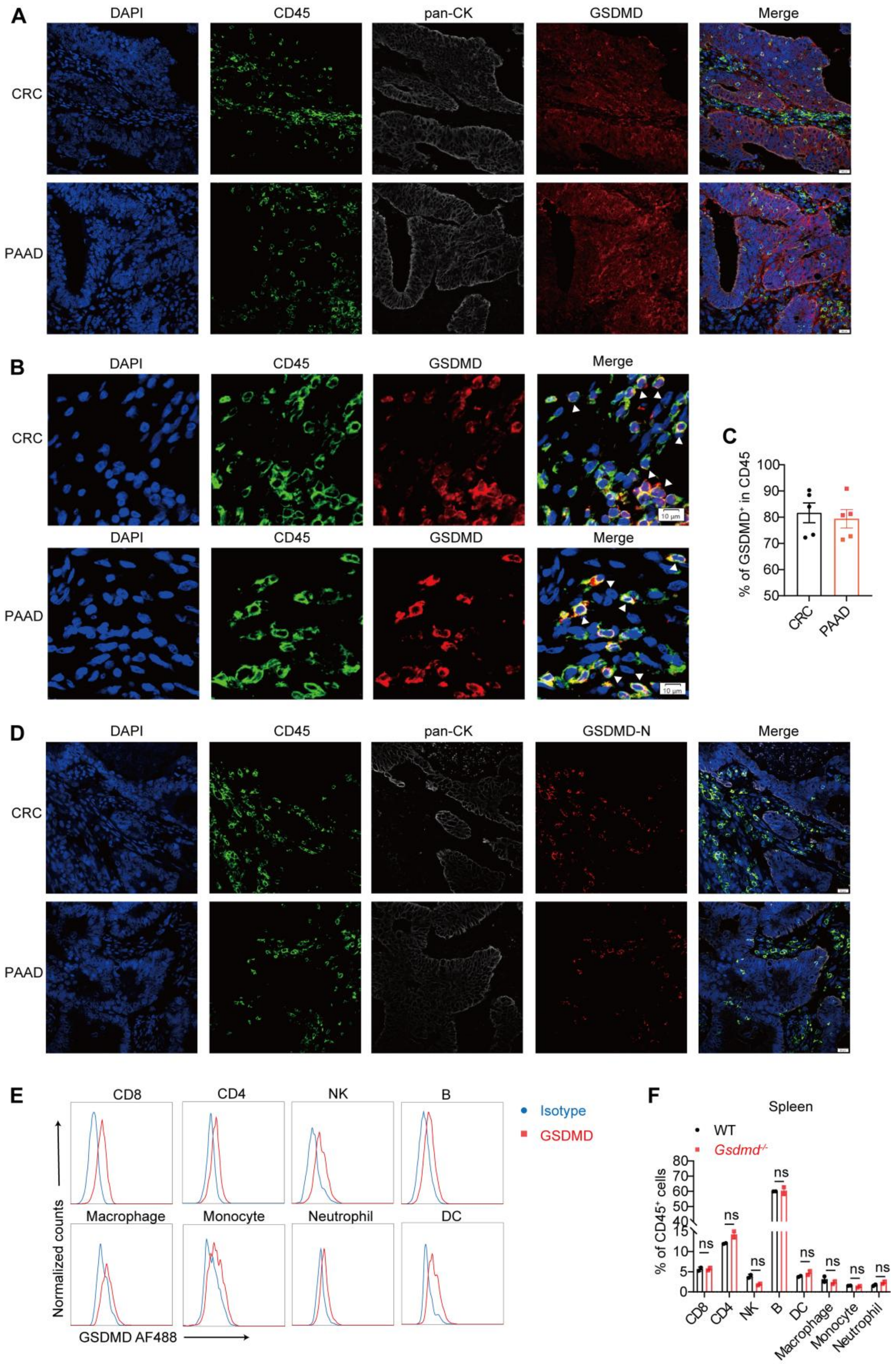

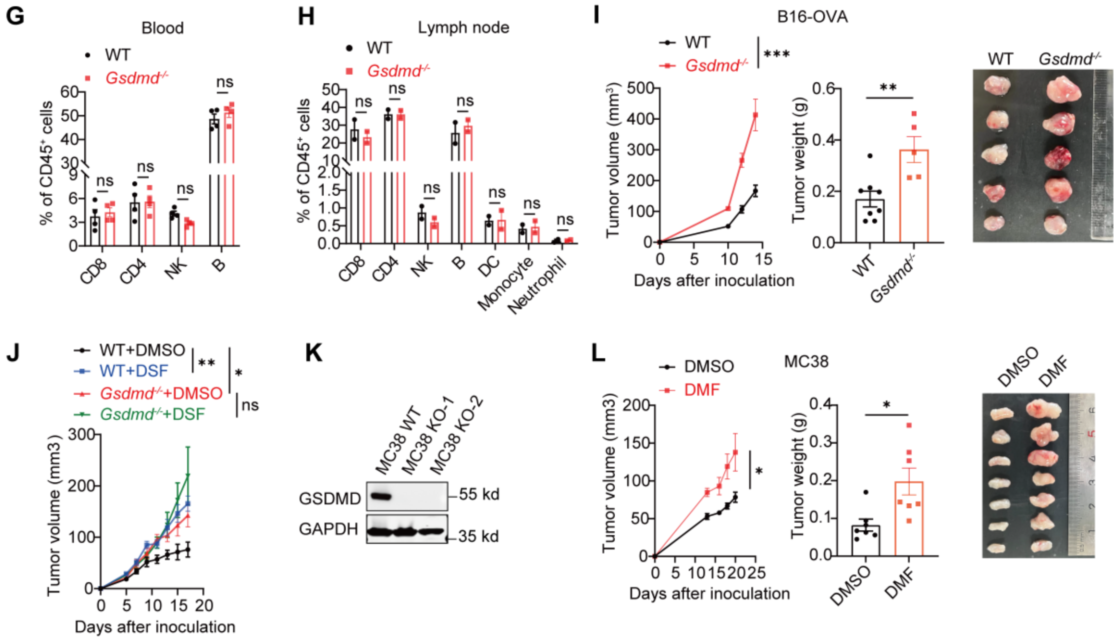

Supplemental Figure 1. GSDMD loss in mice promotes tumor growth.

(A) Immunofluorescence staining of GSDMD (red), CD45 (green) and pan-CK (gray) in tumor tissues from colorectal or pancreatic cancer patients. Scale bars, 20 μm. CRC, colorectal cancer; PAAD, pancreatic adenocarcinoma.

(B and C) Immunofluorescence staining for GSDMD (red) and CD45 (green) in tumor tissues from colorectal or pancreatic cancer patients (**B**). The percentages of GSDMD^{+} cells among CD45^{+} cells were quantified from five independent fields of view within CRC and PAAD tumor tissues (**C**). Scale bars, 10 μm. The white arrowheads indicate GSDMD and CD45 co-expressing cells. CRC, colorectal cancer; PAAD, pancreatic adenocarcinoma.

(D) Immunofluorescence staining of GSDMD-N (red), CD45 (green) and pan-CK (gray) in tumor tissues from colorectal or pancreatic cancer patients. Scale bars, 20 μm. CRC, colorectal cancer; PAAD, pancreatic adenocarcinoma.

(E) Flow cytometry analysis of GSDMD expression in mouse immune cells isolated from spleen.

(F-H) Percentages of immune populations in the spleen (**F**, n=2 per group), blood (**G**, n=4 per group)**,** and lymph nodes (**H**, n=2 per group) of WT and *Gsdmd*$^{-/-}$ mice analyzed by flow cytometry.

(I) Tumor growth curves (left), tumor weights (middle), and representative tumor

images (right) of B16-OVA tumors implanted in WT (n=8) and *Gsdmd*$^{-/-}$ (n=5) mice.

(J) Tumor growth curves of WT and *Gsdmd*$^{-/-}$ mice inoculated with MC38 tumor cells and treated with DMSO or DSF (n=4~5 per group).

(K) Immunoblot analysis of GSDMD protein in WT and GSDMD knockout MC38 cells.

(L) Tumor growth curves of WT and *Gsdmd*$^{-/-}$ mice inoculated with MC38 tumor cells and treated with DMSO or DMF. (n=6~7 per group).

Data are presented as mean ± SEM (**C, F-J, L**) and are representative of at least two independent experiments (**A-J, L**). *$p<0.05$, **$p<0.01$, ***$p<0.001$, ns, not significant, as determined by two-way ANOVA for tumor growth curves or unpaired Student's t-tests for others.

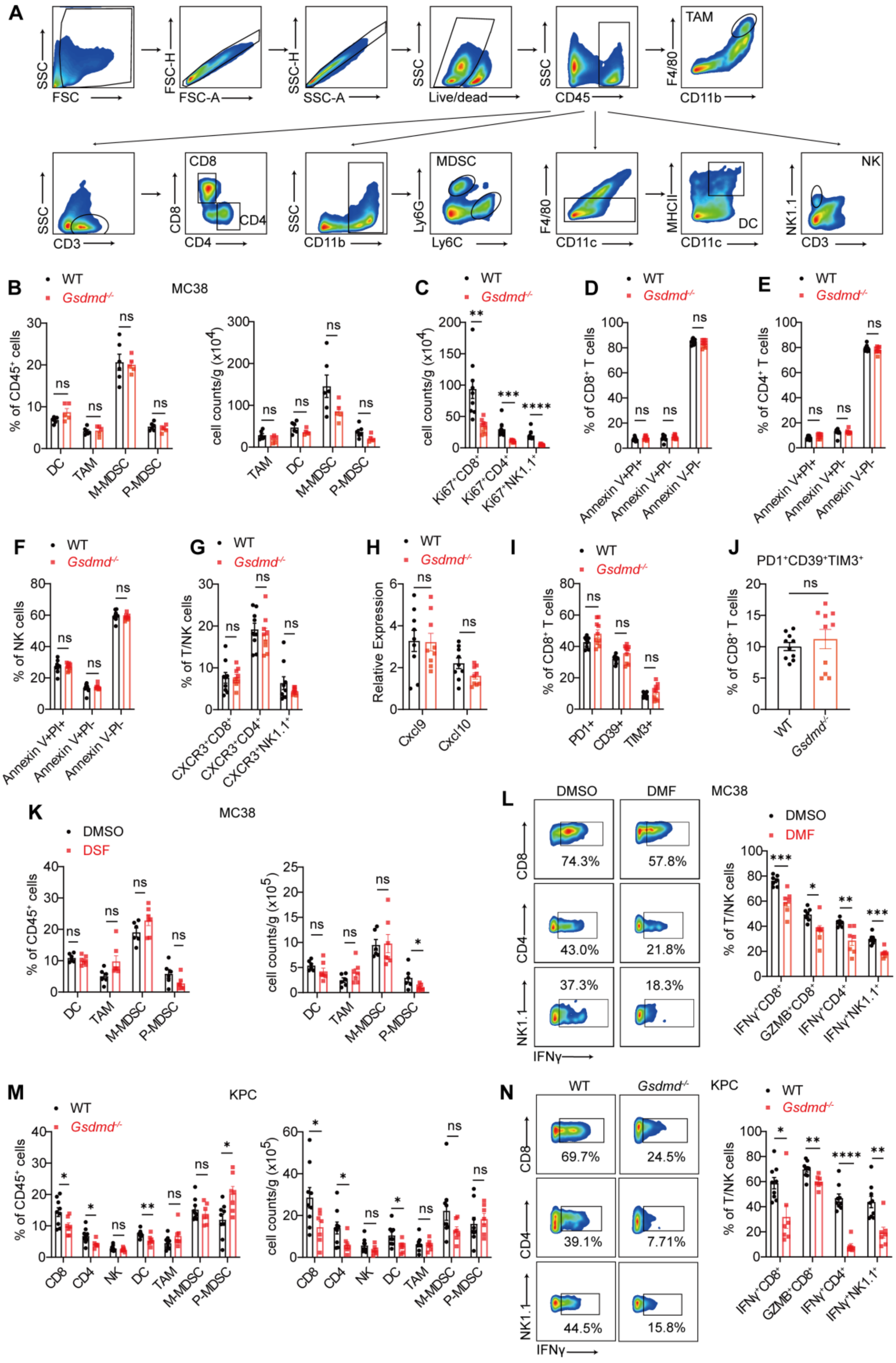
A
TAM
NK
MDSC
DC
B
C
D
E
F
G
H
I
J
PD1⁺CD39⁺TIM3⁺
K
L
M
N
MC38
KPC
WT
Gsdmd-/-
DMSO
DSF
DMF

35

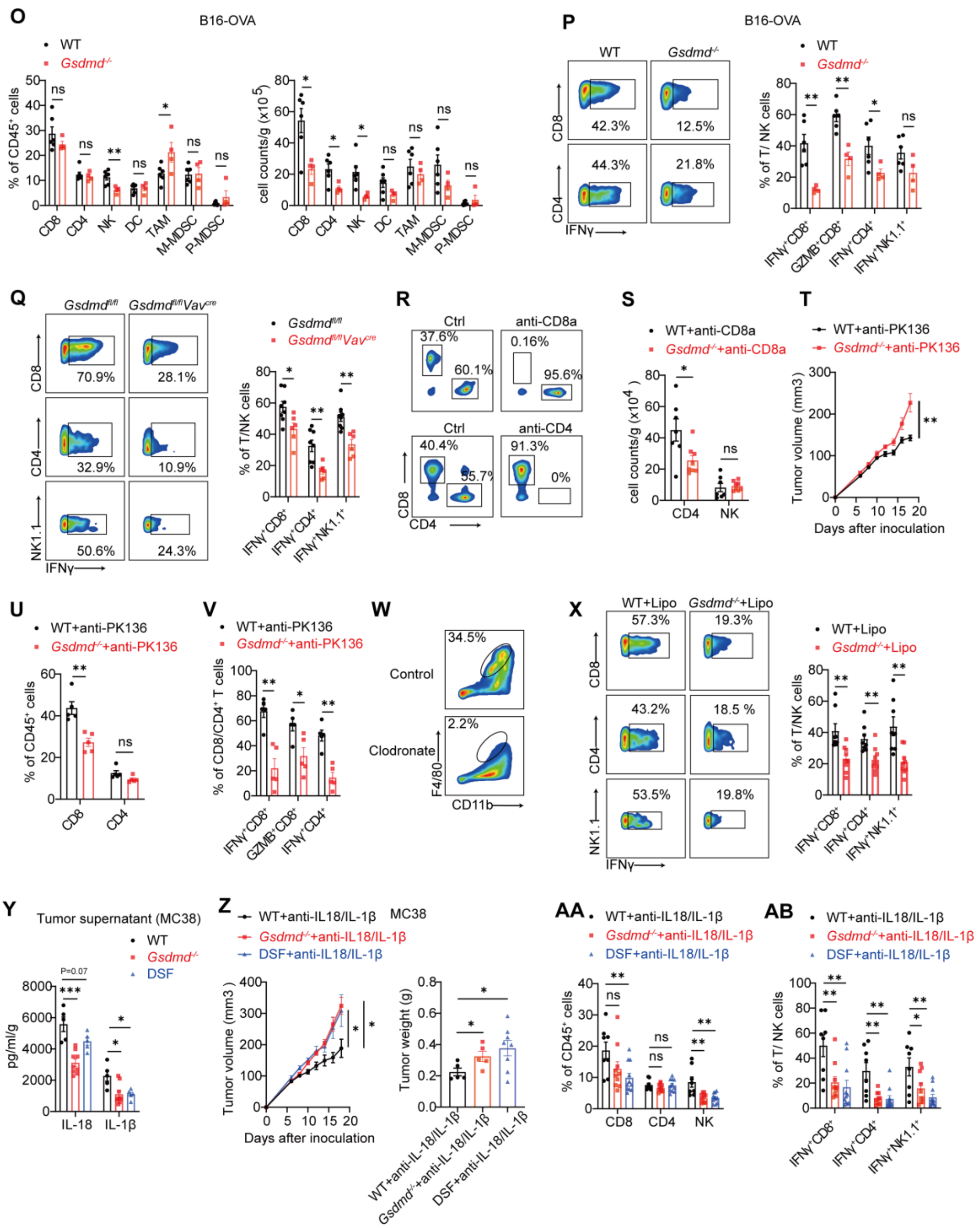

Supplemental Figure 2. GSDMD deficiency impairs T cell-mediated antitumor immunity.

(A) Gating strategy for flow cytometry analysis of tumor-infiltrating immune cells.

(B) Flow cytometry analysis of percentages (left) and cell numbers (right) of immune cells in MC38 tumors implanted in WT (n=6) and *Gsdmd*$^{-/-}$ (n=5) mice.

(C) Flow cytometry analysis of cell numbers of Ki-67-expressing cells in CD8^{+}, CD4^{+} and NK cells in MC38 tumors implanted in WT and *Gsdmd*$^{-/-}$ mice (n=9 per group).

(D-F) Flow cytometry analysis of percentages of Annexin V and PI positive cells in $CD8^+$ (**D**), $CD4^+$ (**E**) and NK cells (**F**) in MC38 tumors implanted in WT and *Gsdmd*$^{-/-}$ mice (n=9 per group).

(G) Flow cytometry analysis of percentages of CXCR3-expressing cells in $CD8^+$ and $CD4^+$ and NK cells in MC38 tumors implanted in WT and *Gsdmd*$^{-/-}$ mice (n=9 per group).

(H) RT-qPCR analysis of CXCL9 and CXCL10 expression in MC38 tumor tissues isolated from WT and *Gsdmd*$^{-/-}$ mice (n=9 per group).

(I and J) Flow cytometry analysis of the expression of PD1, CD39 and TIM3 by $CD8^+$ T cells (**I**) and the percentages of $PD1^+CD39^+TIM3^+CD8^+$ T cells (**J**) in MC38 tumors implanted in WT (n=10) and *Gsdmd*$^{-/-}$ mice (n=10).

(K) Flow cytometry analysis of percentages (left) and cell numbers (right) of immune cells in MC38 tumors implanted in WT mice treated with DMSO (n=6) or DSF (n=7).

(L) Flow cytometry analysis of the percentages of IFN-γ-expressing TILs in MC38 tumors implanted in WT mice treated with DMSO or DMF (n=7 per group).

(M-P) Flow cytometry analysis of immune cell infiltration (**M, O**) and expression of IFN-γ and Granzyme-B by tumor infiltrating lymphocytes (TILs) (**N, P**) in KPC (**M, N**) or B16-OVA tumors (**O, P**) implanted in WT and *Gsdmd*$^{-/-}$ mice. n=9 WT or 7 *Gsdmd*$^{-/-}$ mice in **M, N**; n=6 WT or 4 *Gsdmd*$^{-/-}$ mice in **O, P**.

(Q) Percentages of IFN-γ-expressing TILs in MC38 tumors analyzed by flow cytometry on day 18 after implantation in *Gsdmd*$^{fl/fl}$ (n=8) and *Gsdmd*$^{fl/fl}$*Vav*^{cre} (n=6) mice.

(R) Flow cytometry analysis of percentages of $CD8^+$ T cells and $CD4^+$ T cells in blood of mice treated with CD8α-depleting antibodies or CD4-depleting antibodies.

(S) Cell numbers of $CD4^+$ and NK TILs in MC38 tumors-bearing WT (n=7) and *Gsdmd*$^{-/-}$ (n=8) mice treated with CD8α-depleting antibodies.

(T) Tumor growth curves of MC38 tumors implanted in WT and *Gsdmd*$^{-/-}$ mice and treated with NK-depleting antibodies (PK136) (n=10 per group).

(U and V) Flow cytometry analysis of percentages of $CD8^+$ and $CD4^+$ TILs (**U**) and expression of IFN-γ and Granzyme B by TILs (**V**) in MC38 tumor-bearing WT or

Gsdmd$^{-/-}$ mice treated with NK-depleting antibodies (n=5 per group).

(W and X) Flow cytometry analysis of percentages of tumor-associated macrophages (**W**) and IFN-γ-expressing TILs (**X**) in MC38 tumor-bearing WT (n=8) and *Gsdmd*$^{-/-}$ (n=9) mice treated with control or clodronate liposomes.

(Y) ELISA quantification of IL-18 and IL-1β in supernatant of MC38 tumors implanted in WT (n=5), *Gsdmd*$^{-/-}$ (n=9) or DSF-treated (n=5) mice.

(Z) Tumor growth curves (left) and tumor weights (right) of MC38 tumors implanted in WT (n=5), *Gsdmd*$^{-/-}$ (n=5) and DSF-treated (n=8) mice injected with IL-18 and IL-1β neutralizing antibodies.

(AA and AB) Percentages of $CD8^+$, $CD4^+$ and NK TILs (**AA**) and IFN-γ expression by TILs (**AB**) in MC38 tumors implanted in WT (n=8), *Gsdmd*$^{-/-}$ (n=10) and DSF-treated (n=11) mice injected with IL-18 and IL-1β neutralizing antibodies.

Data are presented as mean ± SEM and are representative of at least two independent experiments (**B-AB**). *$p<0.05$, **$p<0.01$, ***$p<0.001$, ****$p<0.0001$, ns, not significant, as determined by two-way ANOVA for tumor growth curves, one-way ANOVA for Y-AB or unpaired Student's t-tests for others.

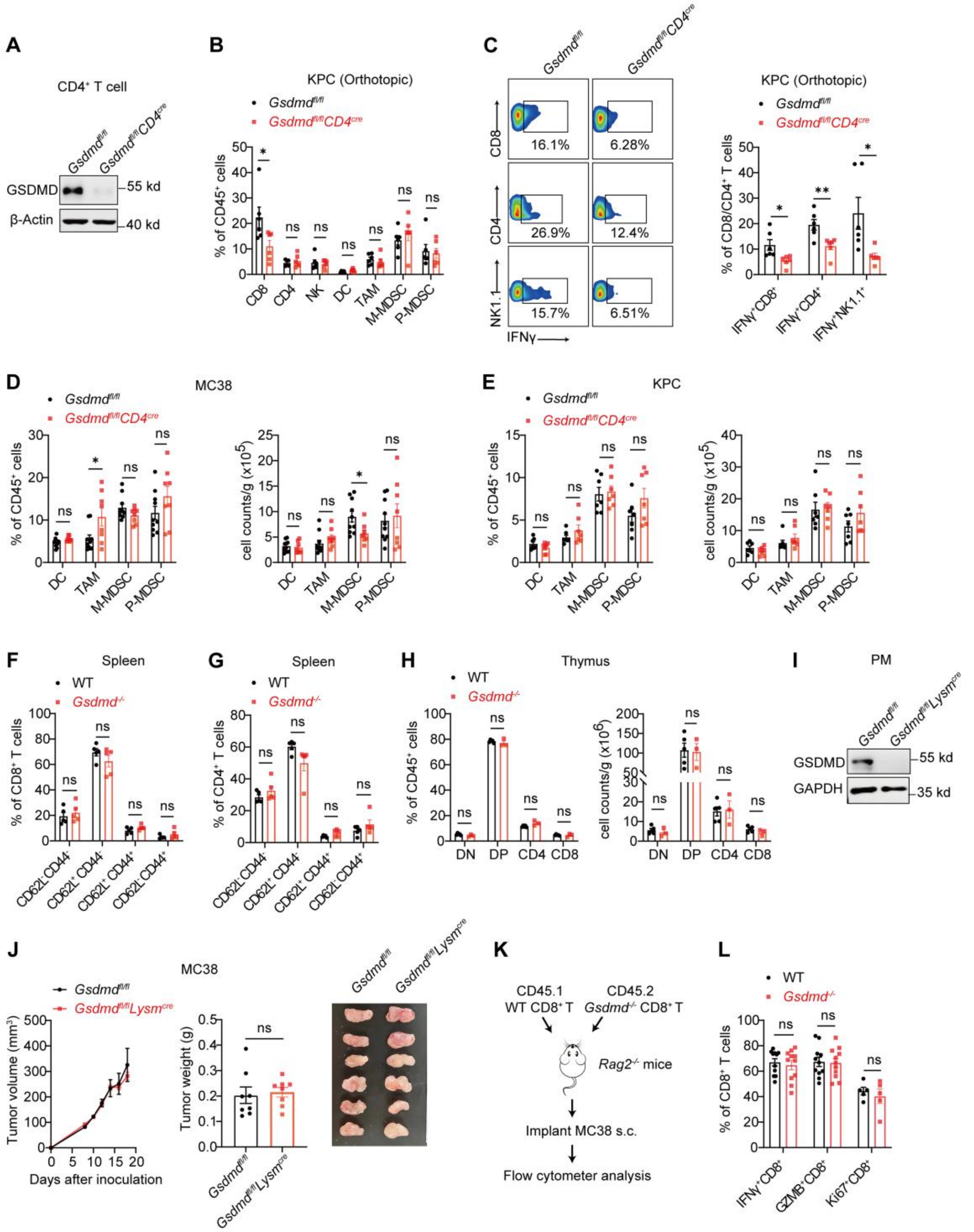

Supplemental Figure 3. GSDMD deficiency in CD4$^+$ T cells leads to impaired CD8$^+$ T cell function.

(A) Immunoblot analysis of GSDMD protein in CD4$^+$ T cells isolated from the spleen of *Gsdmd*$^{fl/fl}$ and *Gsdmd*$^{fl/fl}$*CD4*^{cre} mice.

(B and C) Percentages of immune cells (**B**) and IFN-γ-expressing TILs (**C**) in orthotopic KPC tumors analyzed on day 14 after implantation in *Gsdmd*$^{fl/fl}$ and *Gsdmd*$^{fl/fl}$*CD4*^{cre} mice (n=6 per group).

(D and E) Flow cytometry analysis of percentages (left) and cell numbers (right) of myeloid cells in MC38 (**D**, n=8 or 10 per group) and KPC (**E**, n=7 per group) tumors implanted in $Gsdmd^{fl/fl}$ and $Gsdmd^{fl/fl}CD4^{cre}$ mice.

(F and G) Phenotypic analysis of $CD8^+$ (**F**) and $CD4^+$ (**G**) T cells in spleen of WT and $Gsdmd^{-/-}$ mice based on CD44 and CD62L expression (n=5 per group).

(H) Flow cytometry analysis of thymocytes at different developmental stages (n=5 WT or 3 $Gsdmd^{-/-}$ mice).

(I) Immunoblot analysis of GSDMD protein in peritoneal macrophages isolated from $Gsdmd^{fl/fl}$ and $Gsdmd^{fl/fl}Lysm^{cre}$ mice.

(J) Tumor growth curves (left), tumor weights (middle), and representative tumor images (right) of MC38 tumors implanted in $Gsdmd^{fl/fl}$ and $Gsdmd^{fl/fl}Lysm^{cre}$ mice (n=8 per group).

(K and L) Experimental design (**K**) and flow cytometry analysis (**L**) of effector molecule expression by $CD8^+$ TILs in MC38 tumor-bearing $Rag2^{-/-}$ mice co-transferred with WT CD45.1 and $Gsdmd^{-/-}$ CD45.2 $CD8^+$ T cells (n=11).

Data are presented as mean ± SEM (**B-H**, **J**, **L**) and are representative of at least two independent experiments (**A-L**). *$p<0.05$, **$p<0.01$, ns, not significant, as determined by two-way ANOVA for tumor growth curves or unpaired Student's t-tests for others.

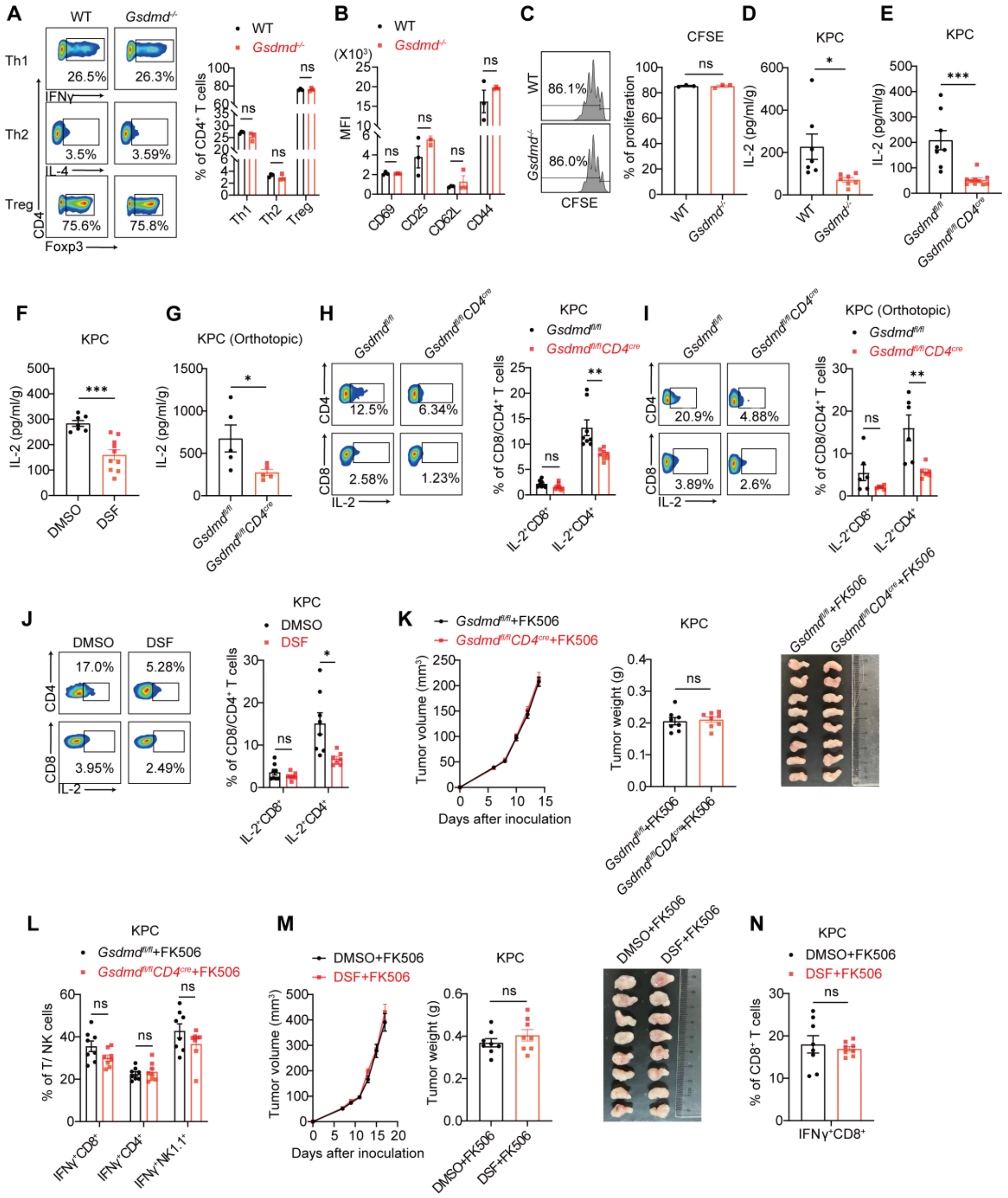

Supplemental Figure 4. CD4$^+$ T cell-intrinsic GSDMD potentiates antitumor immunity via IL-2 induction.

(A) Flow cytometry analysis of Th1 (IFN-γ$^+$CD4$^+$), Th2 (IL-4$^+$CD4$^+$) and Treg (Foxp3$^+$CD4$^+$) differentiation from naive CD4$^+$ T cells isolated from WT and *Gsdmd*$^{-/-}$ mice (n=3 per group).

(B) Flow cytometry analysis of CD69, CD25, CD62L and CD44 expression by CD4$^+$ T cells isolated from WT and *Gsdmd*$^{-/-}$ mice and activated by anti-CD3/CD28 with 10 ng/ml IL-2 in vitro for 72 h.

(C) Flow cytometry analysis of CFSE labeling in $CD4^+$ T cells isolated from WT and *Gsdmd*$^{-/-}$ mice and activated by anti-CD3/CD28 with 10 ng/ml IL-2 in vitro for 72 h.

(D-G) Quantification of IL-2 in supernatant of subcutaneous KPC tumors implanted in WT and *Gsdmd*$^{-/-}$ mice (n=7 per group) (**D**), *Gsdmd*$^{fl/fl}$ (n=8) and *Gsdmd*$^{fl/fl}$*CD4*^{cre} (n=10) mice (**E**), WT mice treated with DMSO (n=7) or DSF (n=10) (**F**), or orthotopic KPC tumor implanted in *Gsdmd*$^{fl/fl}$ and *Gsdmd*$^{fl/fl}$*CD4*^{cre} mice (**G**, n=5 per group).

(H and I) Percentages of IL-2-expressing $CD8^+$ and $CD4^+$ TILs in subcutaneous (**H,** n=8 per group) or orthotopic (**I,** n=6 per group) KPC tumors implanted in *Gsdmd*$^{fl/fl}$ or *Gsdmd*$^{fl/fl}$*CD4*^{cre} mice.

(J) Percentages of IL-2-expressing $CD8^+$ and $CD4^+$ TILs in subcutaneous KPC tumor-bearing mice treated with DMSO (n=8) or DSF (n=7).

(K-N) Tumor growth curves (left), tumor weights (middle), and representative tumor images (right) of KPC tumors implanted in *Gsdmd*$^{fl/fl}$ and *Gsdmd*$^{fl/fl}$*CD4*^{cre} mice (**K**), or DMSO- or DSF-treated WT mice (**M**) under the treatment of FK506. Percentages of IFN-γ-expressing TILs were analyzed by flow cytometry (**L, N,** n=8 per group).

Data are presented as mean ± SEM and are representative of at least two independent experiments (**A-N**). *p<0.05, **p<0.01, ***p<0.001, ns, not significant, as determined by two-way ANOVA for tumor growth curves or unpaired Student's t-tests calculated for others.

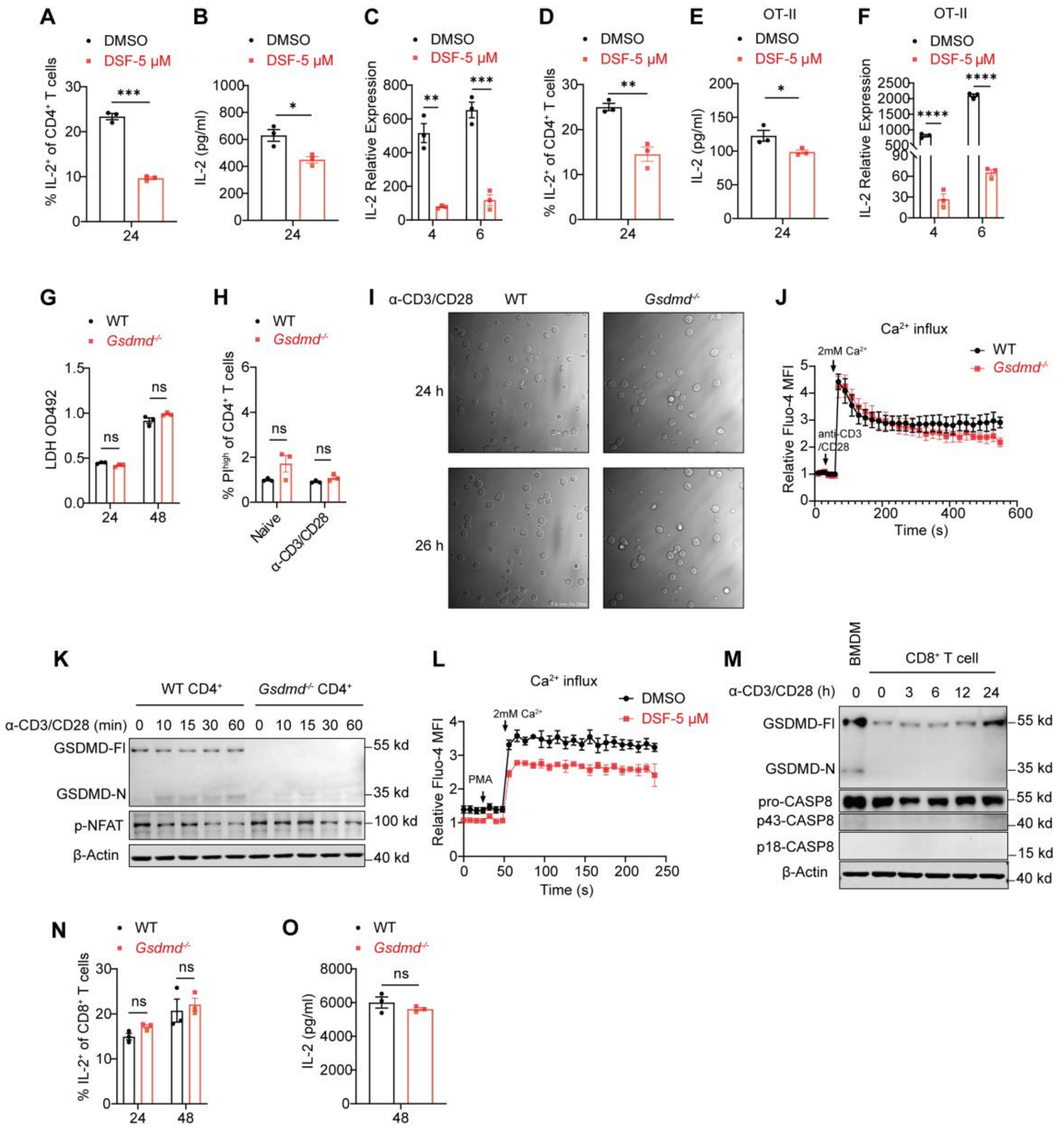

Supplemental Figure 5. GSDMD activation in CD4$^+$ T cells does not induce proptosis.

(A-F) CD4$^+$ T cells isolated from WT or OT-II mice were activated by anti-CD3/CD28 (**A-C**) or OVA$_{323\text{-}339}$ peptide (**D-F**) *in vitro* for the indicated times in the presence or absence of DSF (5 μM). Percentage of IL-2-expressing CD4$^+$ T cells (**A, D**). IL-2 levels in culture supernatants (**B, E**) and IL-2 transcript levels (**C, F**) were assessed. The relative expression level was fold normalized to the IL-2 mRNA level of WT group at 0 h.

(G) The levels of LDH in cell culture supernatants of WT and *Gsdmd*$^{-/-}$CD4$^+$ T cells after *in vitro* activation for 24 h or 48 h, detected by ELISA**.**

(H) Flow cytometry analysis of percentages of PI^{high} $CD4^+$ T cells upon *in vitro* stimulation by anti-CD3/CD28 for 24 h.

(I) Microscopic images of morphological changes of WT and *Gsdmd*$^{-/-}$ $CD4^+$ T cells stimulated by anti-CD3/CD28 for 24 h *in vitro*.

(J) Time-course analysis of Ca^{2+} influx in WT or *Gsdmd*$^{-/-}$ naïve $CD4^+$ T cells in response to anti-CD3/CD28 stimulation.

(K) Immunoblot analysis of GSDMD and NFAT protein activation in WT and *Gsdmd*$^{-/-}$ $CD4^+$ T cells upon anti-CD3/CD28 stimulation for the indicated times.

(L) Time-course analysis of Ca^{2+} influx in *in vitro* activated, DMSO- or DSF-treated $CD4^+$ T cells in response to PMA stimulation.

(M) Immunoblot analysis of GSDMD and caspase-8 protein activation in $CD8^+$ T cells upon anti-CD3/CD28 stimulation for the indicated times. LPS and nigericin co-treated BMDMs were used as the positive control of GSDMD activation.

(N and O) $CD8^+$ T cells isolated from WT and *Gsdmd*$^{-/-}$ mice were activated by anti-CD3/CD28 *in vitro*. Percentages of IL-2-expressing $CD8^+$ T cells (**N**) were analyzed by flow cytometry. Secreted IL-2 was quantified by ELISA (**O**).

Data are presented as mean ± SEM (**A-H**, **J**, **L, N-O,** n=3 per group) and are representative of at least two independent experiments (**A-O**). *p<0.05, **p<0.01, ***p<0.001, ****p<0.0001, ns, not significant, as determined by unpaired Student's t-tests.

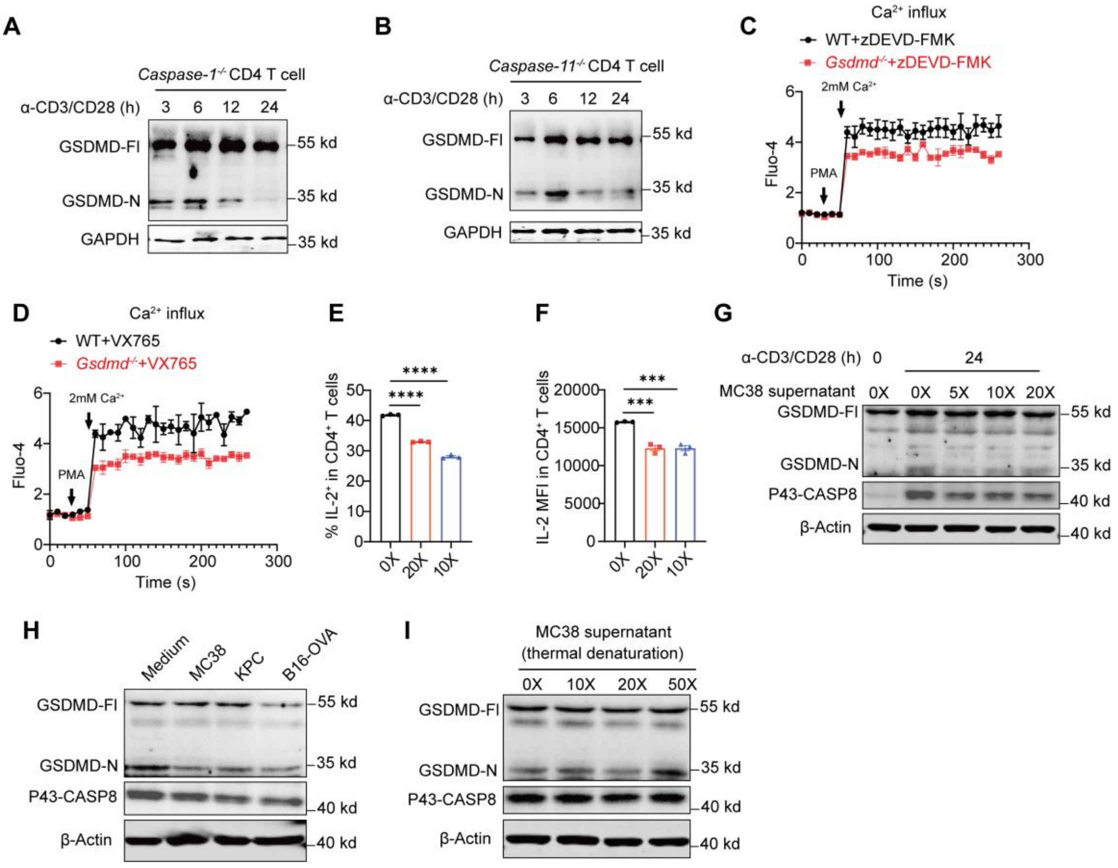

Supplemental Figure 6. Caspase-8 mediates GSDMD activation in $CD4^+$ T cells.

(A and B) Immunoblot analysis of GSDMD protein activation in *caspase-1*$^{-/-}$ (**A**) or *caspase-11*$^{-/-}$ (**B**) $CD4^+$ T cells activated by anti-CD3/CD28 for the indicated times *in vitro*.

(C and D) Time-course analysis of Ca^{2+} influx in *in vitro* activated, inhibitor-treated WT and *Gsdmd*$^{-/-}$ $CD4^+$ T cells in response to PMA stimulation.

(E and F) $CD4^+$ T cells were activated *in vitro* by anti-CD3/CD28 for 24 h with or without MC38 tumor supernatant at different dilutions. Percentages of IL-2 expressing $CD4^+$ T cells (**E**) and IL-2 MFI in $CD4^+$ T cells (**F**) were assessed.

(G-I) Immunoblot analysis of GSDMD and caspase-8 protein activation in activated $CD4^+$ T cells treated with or without the conditioned medium from cultured MC38 cells, KPC and B16-OVA cells (**G, H**), or heat-denatured conditioned medium from MC38 cells (**I**).

Data are presented as mean ± SEM (**C-F**, n=3 per group) and are representative of at least two independent experiments (**C-I**). ***$p<0.001$, ****$p<0.0001$, ns, not

significant, as determined by one-way ANOVA for E and F or unpaired Student's t-tests for others.

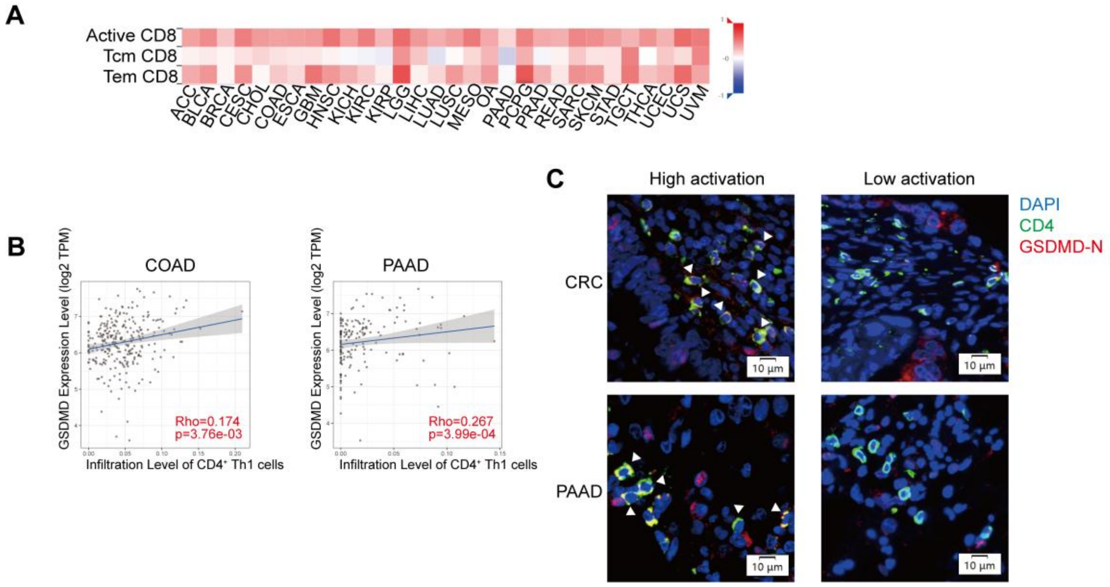

Supplemental Figure 7. GSDMD expression is associated with intratumoral T cell activation in patient samples.

(A) Correlation analysis of GSDMD expression and $CD8^+$ T cell subset infiltration in various types of tumors based on the results from the TISIDB database.

(B) Correlation analysis of GSDMD expression and Th1 infiltration in tumors from patients with COAD or PAAD based on the results from the TIMER database.

(C) Representative images of low- and high- GSDMD activation in tumor-infiltrating $CD4^+$ T cells in colorectal or pancreatic cancer patients.
