# Supplementary material for: A CD4+ T lymphocyte–specific TCR/GSDMD/IL-2 axis facilitates antitumor immunity
Source: J Clin Invest. 2025 Aug 1;135(15):e191119. doi: 10.1172/JCI191119 (PMC12321394; doi:10.1172/JCI191119)
Supplement: Unedited blot and gel images [file jci-135-191119-s179.pdf]

Full unedited blot for Figure 5 D

GSDMD, GSDMD-N

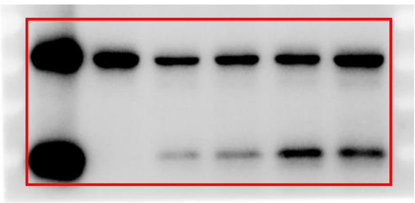

70 kDa
55 kDa
40 kDa
35 kDa

β-Actin

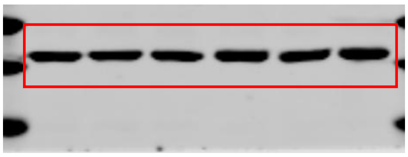

55 kDa
40 kDa
35 kDa

Full unedited blot for Figure 6

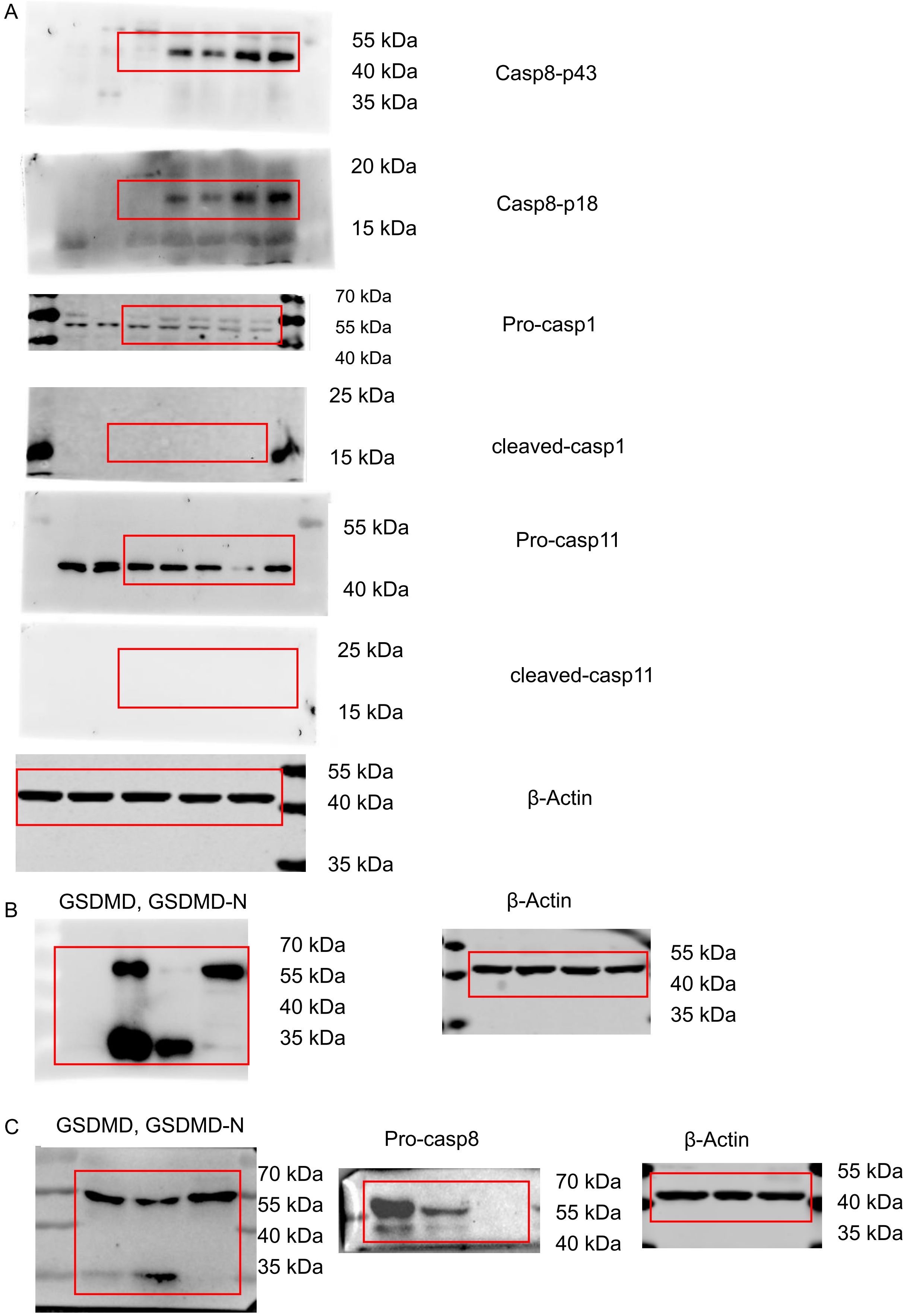

K

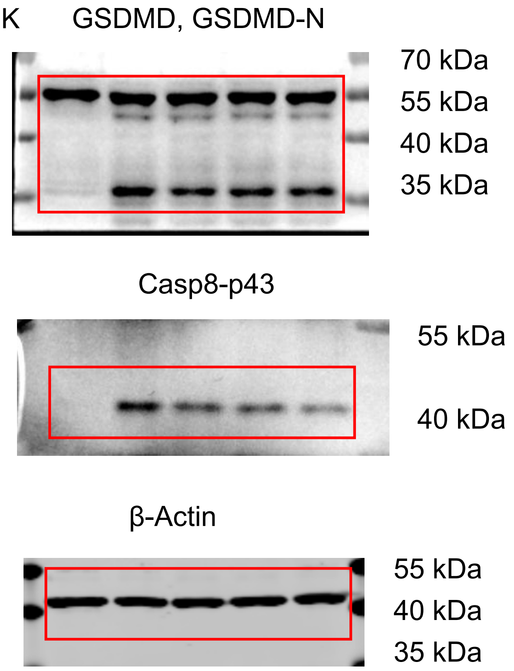
GSDMD, GSDMD-N
70 kDa
55 kDa
40 kDa
35 kDa
Casp8-p43
55 kDa
40 kDa
β-Actin
55 kDa
40 kDa
35 kDa

Full unedited blot for Figure 7A

hGSDMD, hGSDMD-N

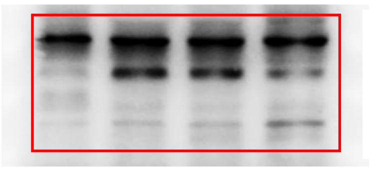

55 kDa
40 kDa
35 kDa

β-Actin

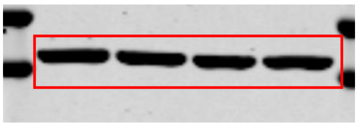

55 kDa
40 kDa
35 kDa

Full unedited blot for Supplementary Figure 1K

GSDMD

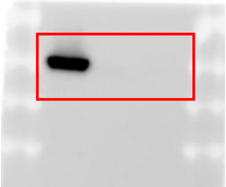

70 kDa
55 kDa
40 kDa
35 kDa

GAPDH

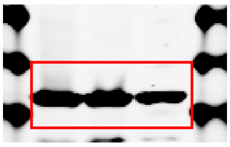

55 kDa
40 kDa
35 kDa

Full unedited blot for Supplementary Figure 3

A

GSDMD

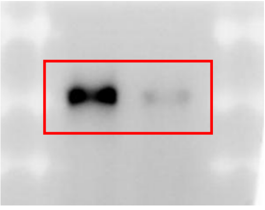

70 kDa
55 kDa
40 kDa

β-Actin

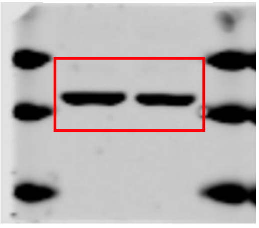

55 kDa
40 kDa
35 kDa

I

GSDMD

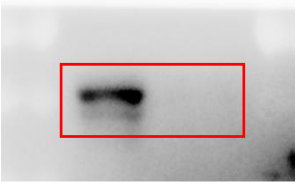

70 kDa
55 kDa
40 kDa

GAPDH

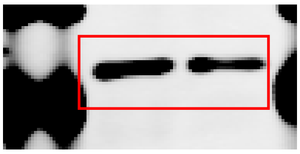

40 kDa
35 kDa

Full unedited blot for Supplementary Figure 5

K

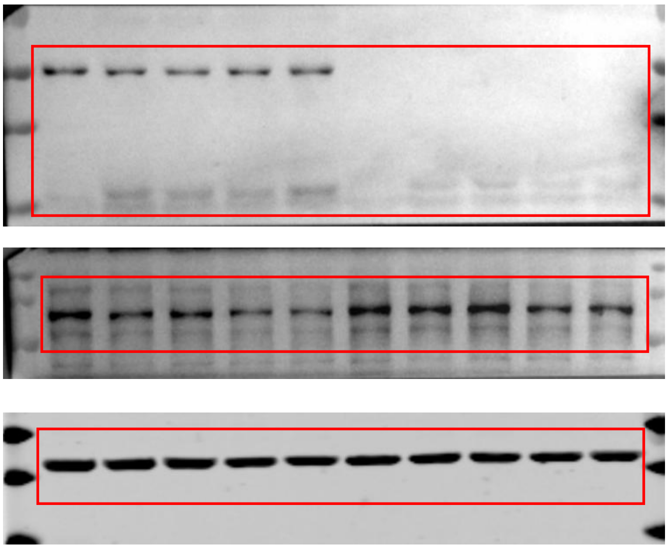

M

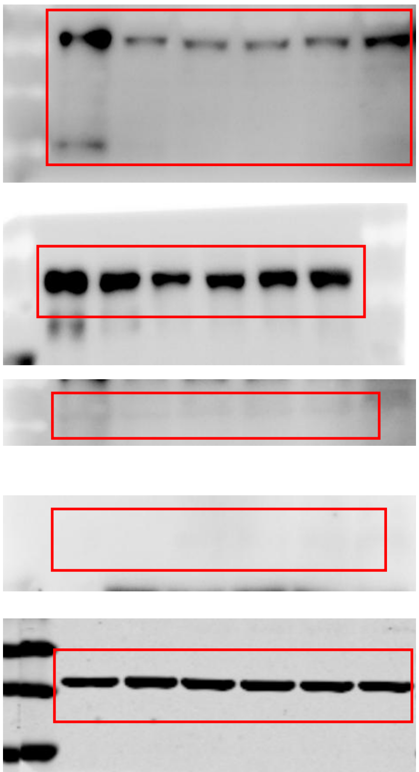

Full unedited blot for Supplementary Figure 6

A

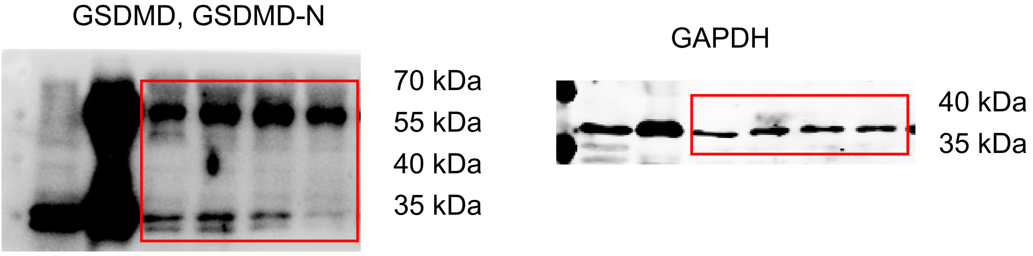

B

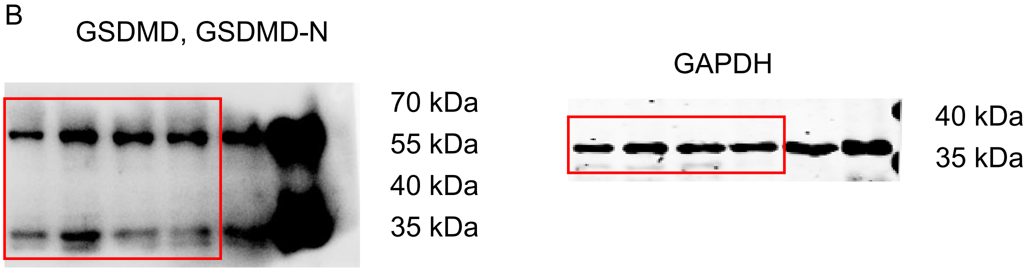

G

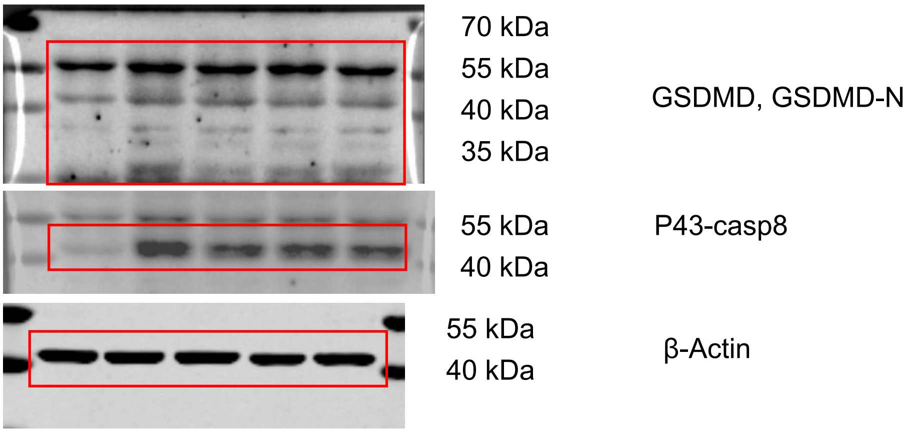

H

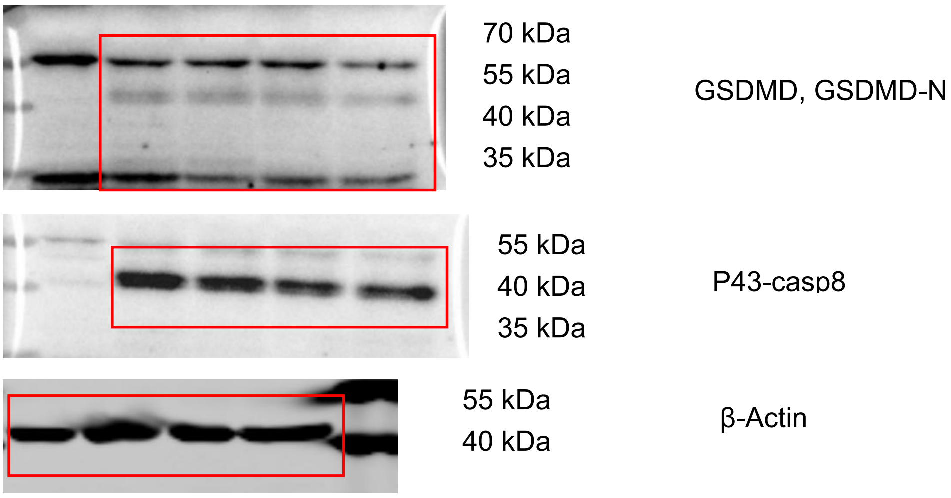

I

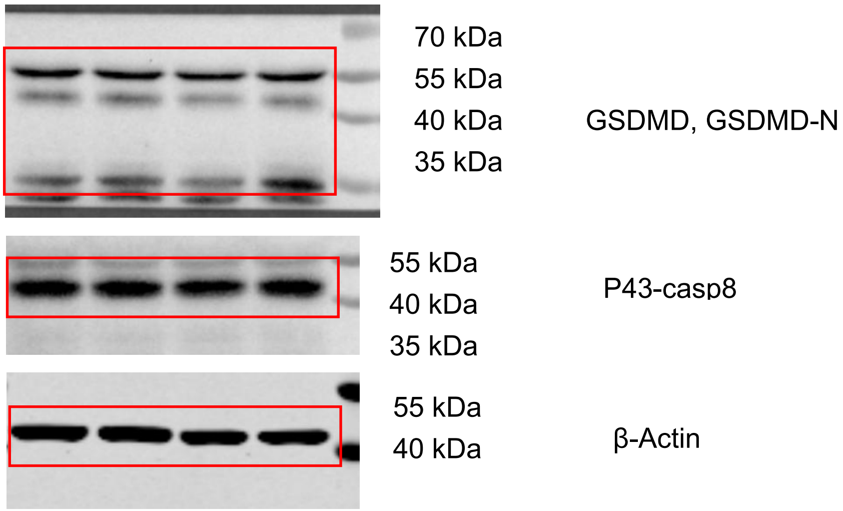
70 kDa
55 kDa
40 kDa
35 kDa
GSDMD, GSDMD-N
55 kDa
40 kDa
35 kDa
P43-casp8
55 kDa
40 kDa
β-Actin
